# (PS)^2^-v2: template-based protein structure prediction server

**DOI:** 10.1186/1471-2105-10-366

**Published:** 2009-10-31

**Authors:** Chih-Chieh Chen, Jenn-Kang Hwang, Jinn-Moon Yang

**Affiliations:** 1 Institute of Bioinformatics, National Chiao Tung University, Hsinchu 30050, Taiwan, Republic of China; 2 Department of Biological Science and Technology, National Chiao Tung University, Hsinchu 30050, Taiwan, Republic of China; 3 Molecular Bioinformatics Center, National Chiao Tung University, Hsinchu 30050, Taiwan, Republic of China

## Abstract

**Background:**

Template selection and target-template alignment are critical steps for template-based modeling (TBM) methods. To identify the template for the twilight zone of 15~25% sequence similarity between targets and templates is still difficulty for template-based protein structure prediction. This study presents the (PS)^2^-v2 server, based on our original server with numerous enhancements and modifications, to improve reliability and applicability.

**Results:**

To detect homologous proteins with remote similarity, the (PS)^2^-v2 server utilizes the S2A2 matrix, which is a 60 × 60 substitution matrix using the secondary structure propensities of 20 amino acids, and the position-specific sequence profile (PSSM) generated by PSI-BLAST. In addition, our server uses multiple templates and multiple models to build and assess models. Our method was evaluated on the Lindahl benchmark for fold recognition and ProSup benchmark for sequence alignment. Evaluation results indicated that our method outperforms sequence-profile approaches, and had comparable performance to that of structure-based methods on these benchmarks. Finally, we tested our method using the 154 TBM targets of the CASP8 (Critical Assessment of Techniques for Protein Structure Prediction) dataset. Experimental results show that (PS)^2^-v2 is ranked 6^th ^among 72 severs and is faster than the top-rank five serves, which utilize *ab initio *methods.

**Conclusion:**

Experimental results demonstrate that (PS)^2^-v2 with the S2A2 matrix is useful for template selections and target-template alignments by blending the amino acid and structural propensities. The multiple-template and multiple-model strategies are able to significantly improve the accuracies for target-template alignments in the twilight zone. We believe that this server is useful in structure prediction and modeling, especially in detecting homologous templates with sequence similarity in the twilight zone.

## Background

For template-based modeling (TBM) and fold recognition methods, a prediction model can be built based on the coordinates of the appropriate template(s) [1]. These approaches generally involve four steps: 1) a representative protein structure database is searched to identify a template that is structurally similar to the protein target; 2) an alignment between the target and the template is generated that should align equivalent residues together as in the case of a structural alignment; 3) a prediction structure of the target is built based on the alignment and the selected template structure, and 4) model quality evaluation. The first two steps significantly affect the quality of the final model prediction in TBM methods.

The secondary structure of a protein is often more conserved than the amino acid sequence, and the prediction accuracy of the secondary structure has been achieved ~80% on average. Recently, a number of methods, integrating secondary structures (i.e., α-helix, β-strand and coil) with primary amino acid sequences, have successfully detected the homologs with remote similarity for automated comparative modeling [2-6] and fold recognition [7-12]. These methods often used two separated substitution matrices [9,10,13] to score secondary structures and primary amino acids, respectively, for aligning a residue pair. The separated matrices are unable to reflect the real score because the amino acid type often prefers to a specific secondary structure.

Here, we have developed a substitution matrix, called S2A2, which considers the properties of the secondary structures and amino acid types. The S2A2 is a 60 × 60 matrix that considers all possible pair combination of 20 amino acid types and three secondary structure elements. This matrix was evaluated on the Lindahl benchmark [14] for fold recognition and the ProSup benchmark [15] for alignment accuracies. According to these evaluation results, the S2A2 matrix has higher accuracy than position specific scoring matrix (PSSM) generated by PSI-BLAST and *prof_sim *for fold recognition and sequence alignments. By integrating the S2A2 matrix and PSSM, each having a unique scoring mechanism, the (PS)^2^-v2 server blends the sequence profile and secondary structure information so that they work cooperatively.

Numerous enhancements and modifications were applied to original (PS)^2 ^servers (namely (PS)^2^-original) [16] and (PS)^2^-CASP8 [17] which participates the CASP8 experiment, thereby improving the reliability and applicability of the method. There are four main differences in methodology between the present server ((PS)^2^-v2) and our previous works (Table 1). First, (PS)^2^-v2 integrates S2A2 matrix and PSSM for the template selection and the target-template alignment to replace a consensus strategy applied in the (PS)^2^-original server. Second, we modified the SSEARCH [18] search method to replace the PSI-BLAST search method and Smith-Waterman algorithm applied in the (PS)^2^-original server and (PS)^2^-CASP8, respectively. Third, (PS)^2^-v2 utilized a new multiple template method for modeling different domains of the target sequence. Finally, (PS)^2^-v2 added a multiple model strategy and utilized ProQ [19] to assess and select the final model. We have assessed the prediction accuracy of the (PS)^2^-v2 server based on the 154 TBM targets of the CASP8 dataset. The experimental results show that the S2A2 matrix, multiple-template and multiple-model strategies are able to significantly improve the accuracies for protein structure prediction and modeling when the sequence similarity between the template and the target is in the twilight zone.

**Table 1:** The essential differences of (PS)^2^-original, (PS)^2^-CASP8 and (PS)^2^-v2

**Steps**	**(PS)^2^-original **[16]	**(PS)^2^-CASP8 **[17]	**(PS)^2^-v2**
1. Template search	Consensus of PSI-BLAST and IMPALA	S2A2+PSSM with a self-developed aligned tool using dynamic programming	S2A2+PSSM with a modified SSEARCH program [18]

2. Target-template alignment	Consensus of PSI-BLAST, IMPALA and T-coffee	S2A2+PSSM with a self-developed aligned tool using dynamic programming	S2A2+PSSM with a modified SSEARCH program [18]

3. Template	Single template	Single template	Multiple templates

4. Model building	MODELLER with single model	MODELLER with single model	MODELLER with multiple models

5. Model evaluation	PROCHECK [42]	PROCHECK	ProQ [19]

## Methods

**Figure 1:**
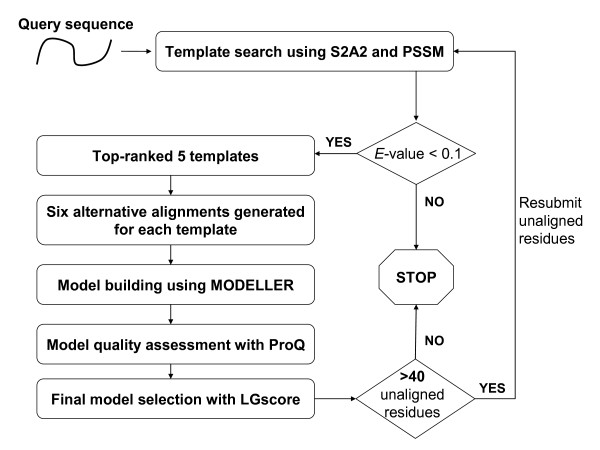
**The framework of the (PS)^2^-v2 server for protein structure prediction**.

**Figure 2:**
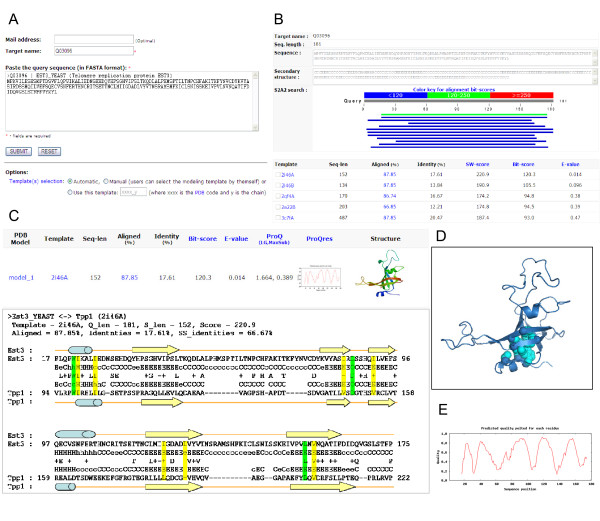
**Overview of the (PS)^2^-v2 server**. The protein sequence of telomere replication protein Est3 (UniProt Q03096) in *Saccharomyces cerevisiae *was used as the query. (A) Input format of the (PS)^2^-v2 server. (B) Search results of a query protein, comprising target name, sequence, predicted secondary structure, the graph of the aligned regions and the hits list of the templates of the query. (C) The selected template, target-template alignment and prediction structure of Est3. (D) The visualization of the predicted structure for Est3. (E) The model quality assessment.

Figures 1 and 2 show the framework of the (PS)^2^-v2 server for protein structure prediction. (PS)^2^-v2 uses the S2A2 matrix and the PSSM for the template selection and the target-template alignment. (PS)^2^-v2 first applied the query sequence to generate a PSSM by running three iterations of PSI-BLAST against a non-redundant sequence UniRef90 [20] with an *E*-value cutoff of 0.001. The PSSM was then used as the input for the PSIPRED [21] tool to predict the secondary structure of this query. We then modified the SSEARCH [18] search method, using the S2A2 matrix and the PSSM as the scoring matrices, to identify the template(s) from the protein structure library, and to generate the target-template alignment(s). The library consists of 20,982 non-redundant structures (April, 2008) selected from protein data bank (PDB) [22]. The secondary structures of each structure in the library were assigned using DSSP [23]. Based on various target-template alignments of top-ranking 5 selected templates, (PS)^2^-v2 generates 30 protein structures using MODELLER [24]. Finally, the program ProQ was used to evaluate these models and to select the final model for the target. The S2A2 matrix, the aligned method, the modeling process and the final model selection are described in the following subsections. The components of the (PS)^2^-v2 server were built using C, Perl and PHP (Additional file 1).

### S2A2 matrix

**Figure 3:**
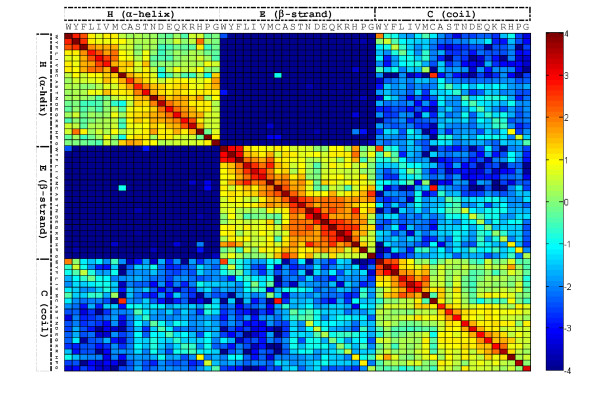
**The S2A2 substitution matrix**. The scores are high if the residue-structure (RS) letters with similar residue types and the same secondary structure are aligned (red blocks). When two identical RS letters (e.g. diagonal entries) are aligned, the substitution scores are very high. In contrast, the scores are low when helix letters are aligned with strand letters (blue blocks).

A substitution matrix is the key component of protein sequence alignment methods. We developed the S2A2 substitution matrix (Figure 3 and Figure S1 in Additional file 2) applying a general mathematical structure [25]. To calculate the S2A2, 674 structural pairs (1,348 proteins) [26], which are structurally similar and with low sequence identity, were selected from SCOP 1.65 [27] based on two criteria: 1) the root-mean-square deviation (rmsd) of a protein pair was be less than 3.5 Å, with more than 70% of aligned residues included in the rmsd calculation, and 2) the sequence identity of a pair is less than 40%. The selected protein pairs had an average sequence identity of 26%, an average rmsd of 2.3 Å and average aligned residues of 90% (207,492 aligned residues out of 230,915 residues). The program DSSP was used to assign the secondary structure for each residue of these 674 structural pairs. The eight types of the secondary structure used in DSSP were reduced to three commonly accepted types (H (helix), E (strand) and C (coil)) according to the following scheme: (H, G, I) → H; (E, B) → E; (T, S, blank) → C. The 20 amino acid types and 3 secondary structure types were converted into 60 residue-structure (RS) types.

The S2A2 matrix (60 × 60) reveals substitution preferences between homologs with low sequence identity, and was developed in a similar way to BLOSUM62 [25] based on these 674 structural pairs. The entry (*S*_*ij*_), which is the substitution score for aligning a RS letter *i*, *j *pair (1 ≤ *i*, *j *≤ 60), of the S2A2 matrix is defined as *S*_*ij *_= λlog_2_(*q*_*ij*_/*e*_*ij*_), where *λ *is a scale factor, and *q*_*ij *_and *e*_*ij *_are the observed and expected probabilities, respectively, of the occurrence of each *i*, *j *pair. The observed probability is given by , where *f*_*ij *_is the total number of aligning *i*, *j *pairs in these 207,492 RS letters. The factor *e*_*ij *_= *p*_*i*_*p*_*j *_if *i *= *j*; otherwise, *e*_*ij *_= 2*p*_*i*_*p*_*j *_(if *i *≠ *j*), where *p*_*i *_is the background probability of occurrence of the letter *i*, and equals . The substitution score is greater than zero (*S*_*ij *_> 0) if the observed probability is greater than the expected probability. By contrast, *S*_*ij *_< 0 if *q*_*ij *_<*e*_*ij*_. The *λ *is optimized by the SALIGN set [28], and is set to 1.6 according to the performance and efficiency.

### Scoring and alignment methods

We modified the SSEARCH program [18], which used a rigorous Smith-Waterman algorithm [29], to search for similarity between a query sequence and template sequences in a library. We optimized the score between the query and template(s) using both S2A2 and PSSM matrices based on alignment accuracies on the SALIGN set. The score is given as



where *i *and *j *are RS letters on the query and the template, respectively; *w*^*stru*^(*i*, *j*) is a structure-dependent scoring weight, and is set to 1.3, 1.7 and 0.8 for α-helix, β-strand and coil, respectively; *w*^*S2A2 *^(here, *w*^*S2A2 *^is set to 0.64) is the weight of the S2A2 matrix; *S2A2*(*i*, *j*) and *PSSM*_*query*_(*i*, *j*) are the scores of S2A2 and PSSM matrices, respectively, when the RS letter *i *is aligned to the RS letter *j*. In addition, we considered structure-dependent gap penalty. Here, *w*^*gap *^is a structure-dependent gapping weight, set to 2.0 (α-helix), 2.0 (β-strand) and 0.15 (coil), respectively; *g*^*S2A2 *^is the gap opening penalty (set to 7.2) and the gap extension (set to 1.2) for the S2A2 matrix. These weights were optimized based on the SALIGN set. *g*^*pssm *^refers to the PSSM, where the gap opening penalty is 11 and gap extension is 1 according to the default parameters of PSI-BLAST.

### Statistics and template selection

SSEARCH provides the statistical significance for library searches. The local sequence similarity score (*S*) follows the extreme value distribution, so that *P*(*S *> x) = 1 - exp(-K*mn *exp(-*λx*)) where *m*, *n *are the lengths of the query and library sequence. The score shows that the average score for an unrelated library sequence increases with the logarithm of the length of the library sequence. SSEARCH uses simple linear regression against the log of the library sequence length to calculate a normalized "z-score" with mean 50, regardless of library sequence length, and variance 10. These z-scores can then be used with the extreme value distribution and the Poisson distribution to calculate the number of library sequences to obtain a score (i.e. *E*-value) greater than or equal to the score obtained in the search. The top-ranking 5 templates with the lowest *E*-values were considered as the templates if the *E*-values < 0.1. For each structure in the top-ranking 5 templates, The (PS)^2^-v2 server generated six alternative target-template alignments by using different S2A2-matrix (w^*S2A2*^) weights, including 0, 0.2, 0.4, 0.64, 0.8 and 1.0. Finally, we yielded 30 target-template alignments for a target protein.

### Model building and evaluation

Protein structure models were built using the homology modeling tool, MODELLER [24] according to the selected template(s) and target-template alignment(s) and then the ability to discriminate a correct protein model from incorrect models is critical when a server used multiple model methods. Here, we utilized the program ProQ [19] to assess the quality of protein models based on the LGscore [30] and a model was considered correct if the LGscore was greater than 1.5 [19]. The (PS)^2^-v2 server first selected the protein model, generated by the first rank template with *w*^*S2A2 *^= 0.64 as the seed model. The LGscore of the seed model was then compared with those of the other models based on the top-rank 5 templates with different w^*S2A2 *^weights. A model was chosen as the final one if it had the highest LGscore and its LGscore (> 0.7) was significantly better than that of the seed model. Otherwise, the server selected the seed model as the final model.

### Multiple-template method

(PS)^2^-v2 considered a target as a multiple domain protein if any region with >40 residues has non-aligned residues to the template(s) when using above "model building and evaluation" steps. For a multiple domain protein, (PS)^2^-v2 automatically decided domain boundaries based on the borders of the large gaps between the target and the template(s), and repeatedly executed above steps to model the structures of the non-aligned residues (Figure 1). Finally, these multiple models were then used as structure templates to generate the full-length final model for the query protein.

## Utility

### Input format

The (PS)^2^-v2 server is an easy-to-use web server (Figure 2). Users input the query protein sequence in FASTA format. The server provides three modes (Automatic, Manual and 'Use this template') for choosing template(s) (Figure 2A). The default mode is 'Automatic'. In this mode, (PS)^2^-v2 automatically selects the modeling template(s). For the 'Manual' mode, our server enables users to assign specific template(s) from a list of candidates (Figure 2B). The 'Use this template' mode allows users to assign a specific protein structure as the template. Finally, (PS)^2^-v2 transmits the predicted results to the users by email addresses.

### Output format

The (PS)^2^-v2 server typically yields a predicted structure within 7 minutes if the query sequence length is ~200. The server shows a list of templates, selected template(s), target-template alignment(s), predicted structure(s) and structure evaluations (Figures 2B and 2C). The predicted structures are visualized in PNG format generated by the MolScript [31] and Raster3D [32] packages. If the user clicks a PNG picture, then the corresponding protein 3D structure is also displayed on the AstexViewer [33] (Figure 2D). A user can download the predicted structure coordinates in the PDB format. The server also provides the target-template alignments and the structure quality factors (Figure 2E).

### Modeling of ever shorter telomeres 3

The ever shorter telomeres 3 (Est3, UniProt Q03096), which is essential for telomere replication *in vivo*, is a small regulatory subunit of telomerase from *Saccharomyces cerevisiae*. According to structure prediction combined with *in vivo *characterization, it has been reported that Est3 consists of a predicted OB-fold (oligosaccharide/oligonucleotide binding) with structurally similar to the OB-fold of the human Tpp1 protein [34]. Because of the limited degree of conservation between these two protein families, these two proteins could not be recognized from simple sequence profile methods. Additionally, the original (PS)^2 ^-v2 server could not recognize them.

For the target Est3, the (PS)^2 ^-v2 server selected the OB-fold domain of the Tpp1 protein (PDB code 2i46) from *Homo sapiens *as the template [35], with an *E*-value of 0.014. This template shared only 17.6% sequence identity with the query sequence. Figure 2C shows the target-template alignment. The server successfully recognized Tpp1 as the template since the secondary structure identity between the template and Est3 was 66.7%. Our method could align together three conserved residues (i.e. Trp21/Trp98, Asp86/Asp148 and Leu155/Leu204, in Est3 versus Tpp1; green blocks in Figure 2C), which are primarily involved in protein folding and/or stability of the OB-fold. Seven amino acid positions (yellow blocks in Figure 2C), which are structurally similar between the two protein families, were also aligned. These 10 aligned residues, depicted in cyan, are clustered in the interior of the core of the OB-fold (Figure 2D).

## Results and Discussion

In the template-based protein structure prediction, the template selection and the target-template alignment are the two critical steps, since they will significantly affect the quality of the final model prediction. The template selections and the sequence alignments of the proposed method with the S2A2 matrix were evaluated by the Lindahl benchmark [14] and ProSup benchmark [15], respectively. In general, it is neither straightforward nor completely fair to compare the results of different fold-recognition and alignment methods given that each employs different sequence databases for sequence profiles, structure databases for structure profiles and properties, release dates, and scoring functions. Therefore, the comparisons between our methods and other published methods serve as an approximate guide. Here, we evaluated S2A2 matrix, PSI-BLAST and *prof_sim *using the same sequence database, UniRef90 [20], with the same parameters to generate a PSSM for fold recognitions (Lindahl benchmark) and sequence alignment (ProSup benchmark). Furthermore, (PS)^2^-v2 was assessed and compared with other 71 automatic servers on 154 TBM targets in CASP8. Please note that (PS)^2^-v2 did not participate in the CASP8 experiment.

### Evaluation of S2A2 matrix

The S2A2 matrix (60 × 60) offers insights about substitution preferences of RS letters between homologous protein sequences (Figure 3 and Figure S1 in Additional file 2). The highest substitution score in this matrix is for the alignment of a RS letter 'W_β_' with a RS letter 'W_β_', where W_β _is the residue Trp with the β-strand structure (Figure S1 in Additional file 2). This substitution score is 6.2. In addition, the substitution scores are also high when two identical structural letters (e.g., diagonal entries) are aligned. For example, the alignment scores are 5.6 and 6.1 while 'W_α_' and 'C_α_' are aligned with 'W_α_' and 'C_β_', respectively; where W_α _is the residue Trp with the α-helix structure and C_α _represents the residue Cys with the α-helix structure. Most of the substitution scores are positive if two RS letters in the same secondary structure are aligned. On the other hand, the lowest substitution score is -7.8 in this S2A2. All of the substitution scores are low when the helix RS letters are aligned with the strand RS letters. The above relationships are in good agreement with biological functions of the relevant structures, showing that the matrix S2A2 embodies conventional knowledge about secondary structure conservation in proteins.

We compared the S2A2 matrix with BLOSUM62. The highest substitution scores are 6.2 (S2A2) and 11 (BLOSUM62). In contrast, the lowest score for S2A2 (-7.8) is much lower than that for BLOSUM62 (-4). The main reasons for this large difference are that α-helices and β-strands constitute very different protein secondary structures, and the RS letters pertaining to these two types of structure are more conserved than amino acid sequences. These results demonstrate that the RS letters with the S2A2 matrix may be able to more accurately find remote homologous sequences than simple amino acid sequence analyses.

### Template selection

For the template selection, our method with S2A2 matrix was compared to other methods on Lindahl benchmark [14], which consists of 976 proteins, for the fold recognition. This set included 555, 434 and 321 assignments for the family, superfamily and fold levels, respectively. The S2A2 matrix outperforms PSI-BLAST and is comparative to other methods on this set (Table 2). Our method (S2A2+PSSM), incorporating PSSM into S2A2, is the best for detecting similarity on the superfamily and fold levels for the top five ranks among the 10 comparative methods. At the superfamily level, the S2A2+PSSM, PSI-BLAST and *prof_sim *[36] identified 75.6%, 49.1% and 61.3% of assignments, respectively. At the fold level, the S2A2+PSSM (54.5%) outperformed PSI-BLAST (14.6%) and *prof_sim *(39.6%) in identifying homologous pairs.

**Table 2:** Comparing S2A2 matrix with other methods for fold recognition on the Lindahl benchmark

**Methods**	**Family (%)**		**Superfamily (%)**		**Fold (%)**	
	
	**Top 1**	**Top 5**	**Top 1**	**Top 5**	**Top 1**	**Top 5**
S2A2 ^a^	77.1	85.1	43.8	63.1	26.5	50.8
S2A2+PSSM ^a^	82.2	88.8	56.7	75.6	27.1	54.5
PSI-BLAST	74.4	79.5	38.5	49.1	4.4	14.6
*prof_sim*	80.7	86.5	50.9	61.3	22.1	39.6
RAPTOR^b^	84.8	87.1	47.0	60.0	31.3	54.2
PROSPECT II^c^	84.1	88.2	52.6	64.8	27.7	50.3
SPARKS^d^	81.6	88.1	52.5	69.1	24.3	47.7
FOLDpro^e^	85.0	89.9	55.5	70.0	26.5	48.3
SP^3 f^	81.6	86.8	55.3	67.7	28.7	47.4
SP^4 f^	80.9	86.3	57.8	68.9	30.8	53.6

^a^This work.

^b, c, d, e, f^Results are summarized from previous works [43,9,45,13], respectively.

### Target-template alignment

For the alignment between the target and the template, our algorithm was evaluated based on the ProSup benchmark [15], which consists of 127 protein pairs with significant structural similarity but with sequence identity of no more than 30%. The total numbers of correctly aligned residue pairs (T_c_) of the S2A2, S2A2+PSSM, *prof_sim *and SSALN [10] were 8732, 9470, 8009 and 9256 pairs, respectively (Table 3). The percentage σ_0 _(average percentage of correctly aligned residues, divided by the length of the structural alignment per protein pair) of the S2A2, S2A2+PSSM, PSI-BLAST, *prof_sim *and SSALN were 53.4%, 58.7%, 36.4%, 43.6% and 58.3%, respectively. The S2A2 matrix is significantly better than those of sequence-based approaches, including FASTA, PSI-BLAST and *prof_sim*. The S2A2+PSSM achieved the highest alignment accuracy with slightly better than SPARKS [9] and SSALN, and much better than the other comparative methods.

**Table 3:** Comparing S2A2 matrix with other methods for sequence alignment accuracies on the ProSup benchmark

**Method**	**T_c_^e^**	**T_m_^e^**	**T_i_^e^**	**σ_0_^e^**
S2A2^a^	8732	947	7198	53.4
S2A2 + PSSM^a^	9470	868	6998	58.7
SSALN^b^	9256	1115	7245	58.3
SPARKS^c^	-	-	-	57.2
*prof_sim*	8009	4505	3142	43.6
PSI-BLAST	6733	4938	3452	36.4
FASTA^d^	5340	3003	7452	31.4

^a^This work.

^b^Results from Qiu and Elber [10].

^c^Results from Zhou and Zhou [9].

^d^Results from Domingues et al. [15].

^e^T_c _and T_m _are total numbers of correctly aligned and missed residue pairs, respectively; T_i _is the total number of incorrect aligned pairs; σ_0 _is the average percentage of correctly aligned residues

### CASP8 structure prediction

**Figure 4:**
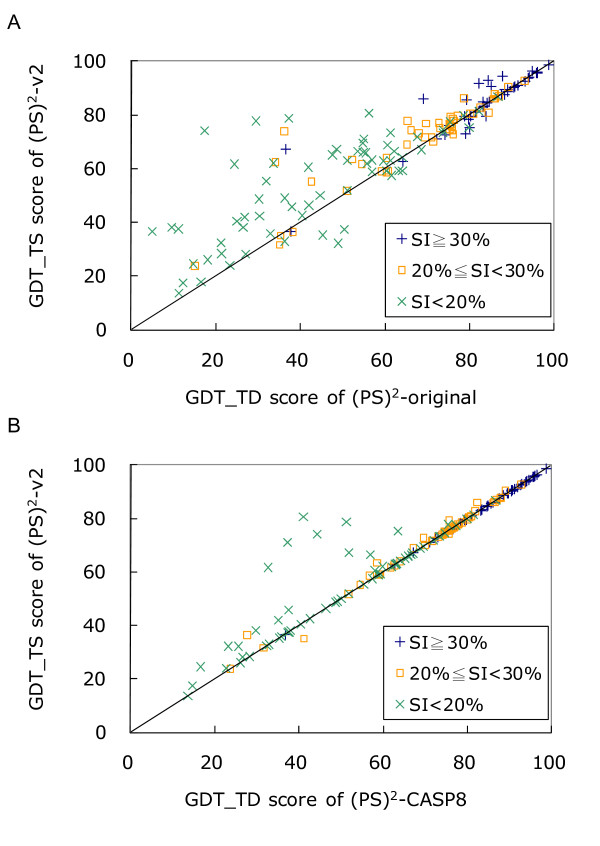
**Comparison the (PS)^2^-v2 server with (A) (PS)^2^-original and (B) (PS)^2^-CASP8 servers on the 154 TBM targets in CASP8**. (PS)^2^-v2 yields 99 and 34 higher GDT_TS scores than (PS)^2^-original and (PS)^2^-CASP8, respectively, among these 154 targets. These three servers have the similar GDT_TS scores when the sequence identity (SI) between the target and template is more than 30% (blue +). (PS)^2^-v2 outperforms our previous servers when SI is less than 20% (green ×).

Our previous server ((PS)^2^-CASP8) and other 70 servers participated in the CASP8 competition, involving 121 targets for tertiary structure prediction. These 121 targets are officially classified into 154 TBM domains (Table S1 in Additional file 3). The accuracies of these 71 servers were evaluated based on the GDT_TS [37] scores directly summarized from the CASP8 website .

(PS)^2^-v2, (PS)^2^-original and (PS)^2^-CASP8 servers were evaluated on these 154 TBM targets (Figure 4, Table 4 and Table S2 in Additional file 4). The sum of GDT_TS scores were 10331.4 ((PS)^2^-v2), 9954.4 ((PS)^2^-CASP8) and 9447.5 ((PS)^2^-original), respectively. (PS)^2^-v2 yielded 99 and 34 higher GDT_TS scores than (PS)^2^-original and (PS)^2^-CASP8, respectively, among 154 targets. When the sequence identity between the target and template was more than 30%, these three servers achieved similar GDT_TS scores. However, if the sequence identity was less than 20%, the (PS)^2^-v2 server was significantly better than (PS)^2^-original server (*p*-value is 4.0E-7) and (PS)^2^-CASP8 (*p*-value is 6.6E-4) using the paired Student's t-test (Table 4). For each target in CASP8, Table S2 (in Additional file 4) shows the GDT_TS score improvement with contributing components (i.e. multiple templates, multiple models, and template search method) between the (PS)^2^-v2 and our previous servers.

These 154 TBM targets were also used to evaluate the automatic servers participating in CASP8. For the templates selection, the accuracy of identifying the best template of the target protein was used to evaluate the performance of these servers (Figure S2 in Additional file 5). The accuracies of the (PS)^2^-v2 server were 54.1% and 75.0% for identifying the Top 1 templates and Top 10 templates, respectively. In addition, (PS)^2^-v2 was the rank 6^th ^among these 72 severs based on GDT_TS scores (Table 5). This server is often able to yield reliable predicted structures (i.e. GDT_TS score = 60%) if the *E*-value is less than 10^-2 ^(Figure S3 in Additional file 6).

The top-rank five serves (Zhang-Server, RAPTOR, pro-sp3-TASSER, Phyre_de_novo and BAKER-ROBETTA) are better than (PS)^2^-v2 on 40 hard targets (i.e., LGA_S score < 70%) (Table S3 in Additional file 7). These serves were much slower than (PS)^2^-v2 because they often utilized *ab initio *methods to build the unaligned loop regions and to generate the models, such as the *Poing *folding system for Phyre_de_novo server, the chunk-TASSER [38] for pro-sp3-TASSER server, and the Rosetta fragment-assembly methodology [39] for BAKER-ROBETTA server. In the near future, our (PS)^2^-v2 server will incorporate *ab initio *methods to model long-length loops and hard targets.

**Table 4:** Comparison the (PS)^2^-v2 server with (PS)^2^-original and (PS)^2^-CASP8 servers on the 154 TBM targets in CASP8 based on GDT_TS scores

**Servers**	**SI^a ^≥ 30% (*n*^b ^= 40)**		**20% ≤ SI < 30% (*n *= 47)**		**SI < 20% (*n *= 67)**	
	
	**Average**	***p*-value**	**Average**	***p*-value**	**Average**	***p*-value**
(PS)^2^-original	82.6	0.0984	67.7	0.0029	44.9	4.0E-7
(PS)^2^-CASP8	84.3	0.323	70.6	0.0766	51.0	6.6E-4
(PS)^2^-v2	84.3	-	71.1	-	54.0	-

^a ^Sequence identity (SI) between the target and the best template.

^b^*n *is the number of targets.

**Table 5:** Comparing (PS)^2^-v2 with 71 automatic servers on 154 targets in CASP8

**Rank**	**Servers**	**Sum of GDT_TS score**
1	Zhang-Server	10870.7
2	RAPTOR	10584.5
3	pro-sp3-TASSER, Phyre_de_novo	10469.3 ~ 10452.9
5	BAKER-ROBETTA, **(PS)^2^-v2**, MULTICOM-CLUSTER	10358.9, **10331.4**, 10325.8
8	METATASSER	10296.7
...	...	...
...	...	...
72	mahmood-torda-server	1355.2

### Multiple templates for multiple domains

**Figure 5:**
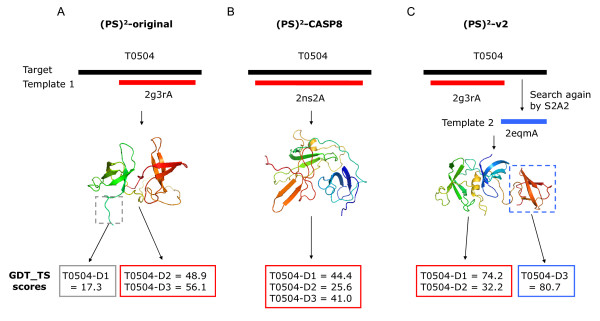
**Comparison the (PS)^2^-v2 server with (PS)^2^-original and (PS)^2^-CASP8 servers on the target T0504 in CASP8**. The (PS)^2^-CASP8 server uses human spindlin1 (PDB code 2ns2) as the template, conversely, (PS)^2^-v2 utilizes a multiple-template strategy and selects both 53BP1 tandem tudor domains (PDB code 2g3r) and PHD finger protein 20-like 1 (PDB code 2eqm) as templates. (PS)^2^-v2 significantly outperforms (PS)^2^-CASP8 on the T0504-D1 and T0504-D3 domains.

We used the target T0504 as an example to describe (PS)^2^-v2 for selecting multiple templates to model protein structures (Figure 5). The (PS)^2^-v2 server first selected the 53BP1 tandem tudor domains (PDB code 2g3r) as the best template. The template 2g3rA aligned a part of regions (138 residues, residues 10-147) to the target, and the model yielded the GDT_TS scores of 74.2 and 32.2 for the target T0504-D1 and T0504-D2. Since the number of the unaligned residues is 61 (residue 148-208), the (PS)^2^-v2 server used unaligned residues to search the new template for modeling this segment. After search template library, (PS)^2^-v2 selected the PHD finger protein 20-like 1 (PDB code 2eqm) as the template for modeling this unmodeling residues (T0504-D3). The GDT_TS score of this model is 80.7 for the target T0504-D3. The total GDT_TS score improvement is 136.42 when (PS)^2^-v2 utilizes a multiple-template strategy. Conversely, the GDT-TS scores of the (PS)^2^-original server, using PDB code 2g3r as the template, are 17.3 (T0504-D1), 48.9 (T0504-D2) and 56.1 (T0504-D3), respectively. For the (PS)^2^-CASP8 server, the GDT-TS scores using PDB code 2ns2 as the template are 44.4 (T0504-D1), 25.6 (T0504-D2) and 41.0 (T0504-D3), respectively.

### Multiple models and model selection

**Figure 6:**
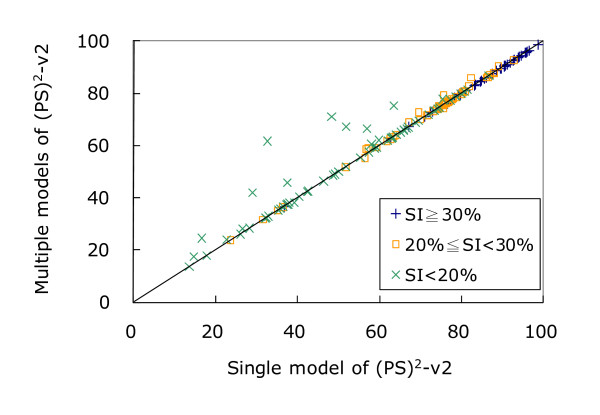
**(PS)^2^-v2 results for using single-model and multiple-model strategies on 154 targets in CASP8 based on GDT_TS scores**. (PS)^2^-v2 improves and decreases the GDT_TS scores for 23 and 4 targets, respectively, when the multiple-model method is utilized. For the other 127 targets, (PS)^2^-v2 obtains the same GDT_TS scores. The symbols "+", "▫" and "×" represent the performance when the sequence identity (SI) ≥ 30%, between 30% and 20%, and less than 20%, respectively.

Figure 6 shows the improvement in GDT_TS scores of (PS)^2^-v2 by applying a multiple-model strategy and using the program ProQ for the final model selection. Among these 154 CASP8 targets, (PS)^2^-v2 improved GDT_TS scores for 23 targets; conversely, only 4 targets are lightly worse when (PS)^2^-v2 used a multiple-model strategy. For the other 127 targets, (PS)^2^-v2 obtained the same GDT_TS scores and the total GDT_TS improvement is 145.3. According to the paired Student's t-test (*p*-value is 0.0045 shown in Table S4 Additional file 8), (PS)^2^-v2 applying the multiple-model strategy significantly improved the GDT_TS scores when the sequence identity between the target and the template is less than 20%.

**Figure 7:**
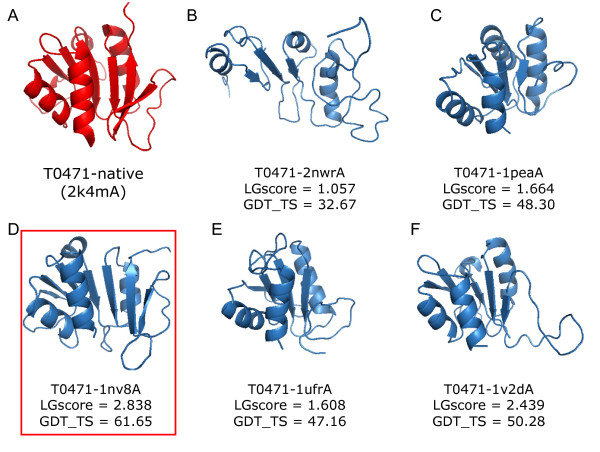
**(PS)^2^-v2 models the target T0471 in CASP 8 using multiple models**. This server models T0471 by selecting top-ranking five structures (PDB code 2nwrA, 1peaA, 1nv8A, 1ufrA and 1v2dA) as templates using S2A2 matrix and PSSM scoring matrices. For each template, (PS)^2^-v2 generates 5 structures and (D) the final model (1nv8) is identified by the program ProQ based on LGscore.

The target T0471 selected from CASP8 was taken as an example to describe the structure modeling of the (PS)^2^-v2 server using multiple-model strategy (Figure 7). When the multiple-model strategy was not considered, (PS)^2^-v2 selected the 2-dehydro-3-deoxyphosphooctonate aldolase (PDB code 2nwr) as the best template with an *E*-value of 0.055. GDT_TS score of this model is 32.67. If we considered the top-ranking 5 structures (PDB codes 2nwr, 1pea, 1nv8, 1ufr and 1v2d) as the modeling templates, (PS)^2^-v2 generated 6 alternative target-template alignments for each template, and obtained 30 alignments for this target. The software MODELLER was then applied to generate 30 structures for these 30 target-template alignments. Figure 7 shows the best model with the highest LGscores, assessing by the program ProQ, for each template. The model generated by the template 1nv8A was selected as the final model, because it had the best LGscore (2.838) among these 30 models. The GDT_TS score of this final model is 61.65. The (PS)^2^-v2 server using multiple models is often able to effectively improve accuracies when the *E*-value between the target and the template is more than 0.01. The average GDT_TS improvements are 8.53 and 2.23, respectively, when the *E*-value ≥ 0.01 and *E*-value ≤ 1e-6.

### T0409 in CASP8

**Figure 8:**
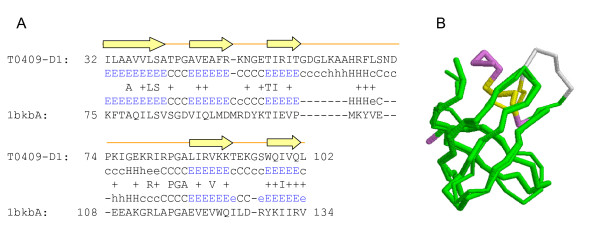
**An example of the prediction results of the target T0409 from the (PS)^2^-v2 server**. The alignment and predicted structure of the BIG_1156.2 domain of putative penicillin-binding protein MrcA from *Nitrosomonas europaea *ATCC 19718 using the (PS)^2^-v2 server. (A) The alignment between the query and the selected template, translation initiation factor 5A protein (PDB code 1bkbA), from *Pyrobaculum aerophilum*. (B) The superposition, the native structure of T0409 (broad, PDB code 3d0f) and the predicted structure (thin). The green blocks are the regions that the predicted structure matches to the native structure. The yellow and purple blocks indicate the shift errors between predicted structure and native structure, the Cα distances between them are <5 Å and >5 Å, respectively.

The target T0409 selected from CASP8 was taken to describe the structure modeling of the (PS)^2^-v2 server (Figure 8). The target is the BIG_1156.2 domain of putative penicillin-binding protein MrcA from *Nitrosomonas europaea *ATCC 19718. This server yielded the best GDT_TS score (77.8) among all participating servers for this target.

For the target T0409, the (PS)^2^-v2 server selected the C-terminal domain of translation initiation factor 5A protein (PDB code 1bkb) from *Pyrobaculum aerophilum *as the template [40]. The C-terminal domain is found to be homologous to the cold-shock protein CspA of *E. coli*, which has a well characterized RNA-binding fold. The best template reported in the CASP8 website is the yeast exosome core, Rrp44 (PDB code 2vnvD) [41], which contains four domains (CSD1, CSD2, RNB and S1). The S1 domain has the most similar structure to the target T0409-D1. The S1 domain also has a common OB fold characteristic of RNA-binding protein, with five anti-parallel β strands. Figure 8A shows the target-template alignment and the template shares 17.0% sequence identity with the query sequence. Our server could align the five anti-parallel β strands together. Figure 8B shows the superposition of the predicted structure (thin) and the X-ray structure (broad) of the target T0409.

## Conclusion

This study presents an automatic server for protein structure predictions by applying numerous enhancements and modifications to the original technique, thereby improving the reliability and applicability. By integrating the S2A2 and PSSM matrixes, the (PS)^2^-v2 server seamlessly blends the amino acid and structural propensities so that they work cooperatively for the template selection and target-template alignments. In addition, our (PS)^2^-v2 utilizes multiple templates and multiple models for building models and assessing models. Experimental results demonstrate that the (PS)^2^-v2 server is efficient and effective for template selections and target-template alignments in template-based modeling. We believe that this server is useful in protein structure prediction and modeling, especially in detecting homologous templates with sequence similarity in the twilight zone.

## Availability and requirements

Project home page: 

Operating system(s): Platform independent

Programming language: C, Perl and PHP

Other requirements: JavaScript-enabled web browser

Any restrictions to use by non-academics: None

## Competing interests

The authors declare that they have no competing interests.

## Authors' contributions

Conceived and designed the experiments: CCC and JMY. Performed the experiments and analyzed the data: CCC and JMY. Contributed reagents/materials/analysis tools and wrote the paper: CCC, JKH and JMY.

## Supplementary Material

Additional file 1 **Program**. The (PS)^2^-v2 program. Click here for file

Additional file 2 **Figure S1**. The S2A2 matrix. Click here for file

Additional file 3 **Table S1**. The summary of 154 TBM targets in CASP8. Click here for file

Additional file 4 **Table S2**. The GDT_TS scores of the (PS)^2^-original, (PS)^2^-CASP8 and (PS)^2^-v2 servers on 154 TBM targets. Click here for file

Additional file 5 **Figure S2**. Comparison of the (PS)^2^-v2 server with top-ranking 45 servers participating in the CASP8 competition for the template selection on 154 TBM targets. The best templates are directly summarized from the CASP8 website . Click here for file

Additional file 6 **Figure S3**. The relation between *E*-values and GDT_TS scores of (PS)^2^-v2 for the targets in CASP8. (PS)^2^-v2 often yields reliable predicted structures if the *E*-value is less than 10^-2^. Click here for file

Additional file 7 **Table S3**. Comparison of the (PS)^2^-v2 server and top five servers in CASP8. Click here for file

Additional file 8 **Table S4**. (PS)^2^-v2 results for using single-model and multiple-model strategies on 154 targets in CASP8 based on GDT_TS scores. Click here for file
