# Supplementary material for: (PS)2-v2: template-based protein structure prediction server
Source: BMC Bioinformatics. 2009 Oct 31;10:366. doi: 10.1186/1471-2105-10-366 (PMC2775752; doi:10.1186/1471-2105-10-366)
Supplement: Additional file 2 — Figure S1. The S2A2 matrix. [file 1471-2105-10-366-S2.pdf]

s_{ij}	A_α	G_α	V_α	R_α	K_α	H_α	D_α	E_α	C_α	N_α	Q_α	I_α	L_α	M_α	P_α	S_α	T_α	F_α	W_α	Y_α	A_β	G_β	V_β	R_β	K_β	H_β	D_β	E_β	C_β	N_β	Q_β	I_β	L_β	M_β	P_β	S_β	T_β	F_β	W_β	Y_β	A_c	G_c	V_c	R_c	K_c	H_c	D_c	E_c	C_c	N_c	Q_c	I_c	L_c	M_c	P_c	S_c	T_c	F_c	W_c	Y_c
A_α	2.6	1.7	14	0.8	09	0.7	04	0.8	16	0.8	1.0	08	0.7	11	0.9	17	1.2	06	0.2	0.5	-5.6	-7.8	-66	-7.8	-51	-7.8	-46	-5.1	-7.8	-59	-6.1	-7.8	-6.8	-55	-5.5	-78	-7.8	-7.0	-78	-5.8	-11	-2.3	-16	-2.0	-21	-1.9	-2.3	-22	-1.9	-22	-2.2	-21	-1.8	-16	-2.4	-1.9	-21	-2.1	-36	-2.3
G_α	1.7	4.4	05	0.6	05	0.7	03	0.4	13	0.9	0.6	-04	-0.3	01	1.0	16	1.1	-02	-0.1	-0.1	-7.8	-3.6	-65	-4.9	-50	-7.8	-45	-7.8	-7.8	-78	-7.8	-78	-6.1	-78	-7.8	-52	-5.5	-7.8	-78	-7.8	-12	-0.4	-22	-1.4	-23	-1.7	-1.7	-17	-1.4	-18	-2.1	-14	-1.9	-25	-1.6	-1.3	-21	-1.9	-20	-2.3
V_α	1.4	0.5	28	0.3	03	0.3	00	0.1	13	-0.1	0.2	24	1.7	16	0.8	07	1.4	15	1.0	0.8	-7.8	-7.8	-73	-7.8	-78	-4.7	-78	-7.8	-4.4	-50	-7.8	-69	-5.3	-78	-7.8	-78	-7.8	-7.8	-44	-7.8	-15	-3.5	-08	-3.1	-27	-3.2	-3.2	-35	-2.5	-31	-1.7	-10	-1.1	-17	-3.1	-2.7	-22	-1.8	-20	-2.9
R_α	0.8	0.6	03	3.4	23	1.5	11	1.4	-06	1.3	1.5	03	0.3	05	0.6	08	0.9	-03	0.6	0.4	-7.8	-5.8	-78	-7.8	-78	-7.8	-41	-5.6	-7.8	-78	-7.8	-78	-5.8	-34	-7.8	-78	-7.8	-7.8	-78	-7.8	-15	-2.5	-27	-0.6	-07	-2.0	-2.2	-16	-3.7	-20	-1.4	-21	-2.2	-27	-2.8	-1.9	-21	-2.4	-18	-2.2
K_α	0.9	0.5	03	2.3	29	1.1	15	1.7	-05	1.5	1.9	02	-0.1	04	0.6	13	1.0	-03	-0.4	0.1	-7.8	-5.0	-74	-7.8	-48	-7.8	-78	-4.8	-7.8	-78	-7.8	-60	-6.9	-78	-4.6	-78	-7.8	-6.2	-78	-7.8	-17	-2.1	-24	-0.8	-06	-2.4	-2.1	-11	-2.3	-13	-1.5	-28	-1.9	-23	-2.2	-1.5	-17	-2.2	-44	-2.4
H_α	0.7	0.7	03	1.5	11	4.5	10	1.1	03	1.7	1.5	05	0.2	09	-0.2	08	1.1	10	0.6	2.0	-7.8	-7.8	-78	-7.8	-42	-2.1	-37	-7.8	-7.8	-78	-7.8	-78	-7.8	-78	-3.0	-44	-7.8	-4.5	-78	-4.3	-18	-1.9	-17	-1.2	-19	0.9	-2.0	-25	-3.2	-18	-1.5	-10	-2.5	-48	-2.6	-2.7	-20	-1.5	-27	-1.3
D_α	0.4	0.3	00	1.1	15	1.0	36	2.1	-09	1.9	1.7	-05	-0.6	-07	1.0	13	1.2	-06	-0.2	-0.1	-7.8	-4.6	-70	-5.4	-78	-7.8	-39	-5.4	-7.8	-47	-7.8	-78	-5.0	-42	-4.2	-56	-4.4	-4.8	-78	-7.8	-12	-2.0	-31	-1.7	-14	-1.0	-0.5	-12	-2.9	-09	-1.3	-20	-2.2	-19	-1.9	-1.9	-20	-2.1	-17	-2.2
E_α	0.8	0.4	01	1.4	17	1.1	21	3.0	-05	1.3	2.0	-04	-0.2	03	1.0	11	1.1	-04	-0.3	0.0	-7.8	-4.8	-68	-7.8	-78	-7.8	-57	-6.2	-7.8	-78	-7.8	-58	-7.4	-78	-7.8	-78	-7.8	-6.6	-78	-7.8	-18	-2.0	-25	-2.5	-19	-1.8	-1.2	-04	-3.7	-17	-1.4	-29	-2.6	-30	-2.3	-1.5	-21	-2.4	-28	-2.8
C_α	1.6	1.3	13	-0.6	-05	0.3	-09	-0.5	61	0.7	0.2	09	0.9	13	-0.9	12	1.1	06	0.4	0.9	-7.8	-7.8	-78	-7.8	-78	-7.8	-78	-7.8	-1.0	-78	-7.8	-78	-4.5	-78	-7.8	-78	-7.8	-7.8	-78	-7.8	-29	-2.6	-30	-2.0	-27	-3.1	-3.8	-26	2.6	-34	-3.5	-36	-1.9	-48	-4.8	-2.1	-29	-4.8	-19	-4.8
N_α	0.8	0.9	-01	1.3	15	1.7	19	1.3	07	3.8	1.5	-03	0.0	04	0.2	14	1.5	02	0.2	0.3	-5.3	-4.0	-48	-7.8	-48	-3.8	-78	-4.8	-7.8	-78	-7.8	-60	-5.9	-78	-3.6	-40	-4.4	-5.2	-78	-7.8	-12	-2.2	-23	-1.0	-08	-1.7	-1.4	-11	-2.3	-03	-0.8	-31	-2.2	-29	-2.8	-1.7	-20	-1.6	-34	-1.0
Q_α	1.0	0.6	02	1.5	19	1.5	17	2.0	02	1.5	3.0	01	0.2	08	0.7	12	1.3	-02	0.2	0.5	-5.8	-7.8	-78	-5.3	-53	-3.3	-48	-5.3	-7.8	-78	-7.8	-78	-5.4	-78	-7.8	-78	-3.9	-4.7	-78	-7.8	-14	-2.1	-34	-1.0	-13	-1.7	-2.3	-13	-2.8	-10	-0.7	-21	-1.9	-14	-2.4	-2.2	-20	-2.6	-19	-2.7
I_α	0.8	-0.4	24	0.3	02	0.5	-05	-0.4	09	-0.3	0.1	31	2.3	21	0.2	03	0.8	16	0.7	0.8	-7.8	-5.8	-78	-5.6	-78	-7.8	-78	-7.8	-7.8	-78	-7.8	-68	-7.8	-78	-7.8	-78	-7.8	-4.4	-43	-7.8	-28	-3.5	-15	-4.3	-29	-2.1	-4.3	-34	-2.1	-27	-2.3	-10	-0.9	-07	-3.4	-4.0	-24	-1.8	-12	-2.8
L_α	0.7	-0.3	17	0.3	-01	0.2	-06	-0.2	09	0.0	0.2	23	2.8	21	-0.2	00	0.8	18	1.1	1.0	-6.2	-7.8	-72	-7.8	-51	-5.6	-78	-7.8	-7.8	-59	-6.1	-78	-6.2	-78	-5.5	-58	-5.6	-6.0	-53	-5.8	-17	-3.0	-19	-2.5	-24	-2.1	-2.9	-22	-2.1	-24	-2.4	-12	-0.2	-07	-3.1	-2.7	-27	-0.8	-16	-1.9
M_α	1.1	0.1	16	0.5	04	0.9	-07	0.3	13	0.4	0.8	21	2.1	36	0.1	05	1.0	15	1.1	0.9	-7.8	-7.8	-78	-7.8	-44	-3.4	-78	-7.8	-7.8	-78	-7.8	-78	-5.5	-78	-7.8	-78	-7.8	-7.8	-78	-7.8	-15	-4.5	-16	-1.9	-19	-2.1	-3.5	-19	-4.8	-20	-3.0	-15	-2.1	02	-3.2	-2.0	-18	-0.9	-06	-1.5
P_α	0.9	1.0	08	0.6	06	-0.2	10	1.0	-09	0.2	0.7	02	-0.2	01	4.9	12	1.1	-06	-0.3	0.2	-4.9	-7.8	-78	-7.8	-78	-7.8	-39	-7.8	-3.1	-78	-7.8	-56	-4.5	-32	-7.8	-78	-7.8	-4.8	-78	-4.5	-08	-1.5	-13	-1.2	-10	-2.1	-1.8	-11	-4.8	-15	-1.1	-15	-2.2	-19	0.6	-0.8	-18	-1.9	-20	-1.8
S_α	1.7	1.6	07	0.8	13	0.8	13	1.1	12	1.4	1.2	03	0.0	05	1.2	27	1.9	00	0.3	0.3	-4.3	-5.6	-69	-5.4	-38	-7.8	-78	-7.8	-7.8	-37	-7.8	-66	-7.8	-78	-7.8	-40	-6.0	-7.8	-78	-5.5	-09	-1.7	-19	-1.3	-11	-1.8	-1.6	-11	-2.1	-11	-1.3	-23	-1.7	-22	-1.9	-0.5	-14	-1.9	-30	-1.4
T_α	1.2	1.1	14	0.9	10	1.1	12	1.1	11	1.5	1.3	08	0.8	10	1.1	19	3.0	03	0.2	0.3	-5.7	-7.8	-52	-7.8	-78	-7.8	-78	-7.8	-3.9	-78	-7.8	-64	-7.8	-40	-7.8	-78	-4.8	-7.8	-29	-5.3	-14	-2.4	-15	-2.1	-16	-2.0	-2.7	-23	-1.5	-18	-1.4	-23	-1.9	-27	-2.5	-1.2	-07	-2.0	-18	-1.6
F_α	0.6	-0.2	15	-0.3	-03	1.0	-06	-0.4	06	0.2	-0.2	16	1.8	15	-0.6	00	0.3	39	2.3	2.6	-5.7	-7.8	-57	-7.8	-52	-7.8	-78	-5.2	-7.8	-78	-7.8	-53	-7.8	-40	-7.8	-78	-7.8	-4.5	-39	-7.8	-29	-2.6	-19	-2.5	-24	-1.9	-2.4	-25	-2.2	-27	-3.8	-15	-0.7	-15	-4.0	-3.3	-28	0.4	-12	-0.9
W_α	0.2	-0.1	10	0.6	-04	0.6	-02	-0.3	04	0.2	0.2	07	1.1	11	-0.3	03	0.2	23	5.6	2.7	-7.8	-7.8	-78	-7.8	-78	-7.8	-78	-7.8	-7.8	-78	-7.8	-49	-7.8	-78	-7.8	-78	-7.8	-4.1	-24	-7.8	-19	-3.3	-12	-1.6	-27	-0.9	-2.6	-23	-1.2	-25	-2.3	-20	-1.7	-05	-2.6	-2.5	-33	-0.9	19	-0.7
Y_α	0.5	-0.1	08	0.4	01	2.0	-01	0.0	09	0.3	0.5	08	1.0	09	0.2	03	0.3	26	2.7	4.3	-7.8	-5.1	-64	-7.8	-78	-3.8	-78	-4.9	-7.8	-78	-4.3	-78	-7.8	-78	-3.7	-51	-5.4	-7.8	-78	-5.0	-22	-3.1	-15	-2.9	-28	-1.6	-1.7	-17	-1.9	-21	-1.2	-13	-1.1	-14	-2.4	-2.3	-21	-0.4	00	0.5
A_β	-5.6	-7.8	-78	-7.8	-78	-7.8	-78	-7.8	-78	-5.3	-5.8	-78	-6.2	-78	-4.9	-43	-5.7	-57	-7.8	-7.8	38	2.5	18	1.1	07	1.2	08	0.9	2.3	11	1.6	13	1.2	20	1.5	22	1.7	1.1	09	1.2	-07	-1.6	-10	-2.5	-22	-2.1	-2.7	-26	-2.7	-26	-1.9	-23	-2.2	-33	-1.6	-1.7	-16	-2.0	-38	-2.0
G_β	-7.8	-3.6	-78	-5.8	-50	-7.8	-46	-4.8	-78	-4.0	-7.8	-58	-7.8	-78	-7.8	-56	-7.8	-78	-7.8	-5.1	25	5.0	01	0.5	04	0.6	04	0.2	1.4	15	1.2	00	-0.1	05	0.4	19	0.9	0.3	03	0.8	-13	0.3	-17	-1.4	-19	-0.6	-1.7	-17	-1.6	-16	-2.5	-23	-2.7	-20	-2.1	-1.4	-23	-3.4	-34	-2.2
V_β	-6.6	-6.5	-73	-7.8	-74	-7.8	-70	-6.8	-78	-4.8	-7.8	-78	-7.2	-78	-7.8	-69	-5.2	-57	-7.8	-6.4	18	0.1	32	0.5	09	0.7	-01	0.7	1.7	01	0.8	27	2.1	20	1.0	10	1.4	1.5	09	1.1	-20	-3.2	-08	-2.3	-30	-2.3	-3.1	-30	-2.3	-32	-2.9	-12	-1.3	-18	-2.1	-2.6	-20	-2.7	-18	-2.7
R_β	-7.8	-4.9	-78	-7.8	-78	-7.8	-54	-7.8	-78	-7.8	-5.3	-56	-7.8	-78	-7.8	-54	-7.8	-78	-7.8	-7.8	11	0.5	05	4.4	32	2.1	17	2.2	-0.5	17	2.5	04	0.9	09	0.5	17	1.7	0.8	03	1.1	-17	-2.4	-17	0.4	-08	-2.1	-2.0	-06	-2.7	-16	-1.6	-15	-2.0	-28	-1.4	-2.2	-10	-1.5	-16	-1.8
K_β	-5.1	-5.0	-78	-7.8	-48	-4.2	-78	-7.8	-78	-4.8	-5.3	-78	-5.1	-44	-4.4	-38	-7.8	-52	-7.8	-7.8	07	0.4	09	3.2	41	1.7	17	2.6	0.3	19	2.5	05	0.7	07	1.5	20	2.0	0.2	09	1.1	-14	-2.3	-16	-0.7	01	-1.6	-1.8	-07	-1.7	-13	-1.0	-34	-2.0	-22	-1.4	-1.4	-09	-2.5	-13	-1.6
H_β	-7.8	-7.8	-47	-7.8	-78	-2.1	-78	-7.8	-78	-3.8	-3.3	-78	-5.6	-34	-7.8	-78	-7.8	-78	-7.8	-3.8	12	0.6	07	2.1	17	5.4	20	1.9	0.5	27	2.5	06	0.4	11	1.3	18	1.3	1.5	17	2.0	-32	-2.7	-23	-1.7	-16	1.1	-2.5	-08	-1.1	-22	-1.2	-19	-1.9	-27	-1.7	-1.8	-13	-1.8	-48	-1.3
D_β	-4.6	-4.5	-78	-4.1	-78	-3.7	-39	-5.7	-78	-7.8	-4.8	-78	-7.8	-78	-3.9	-78	-7.8	-78	-7.8	-7.8	08	0.4	-01	1.7	17	2.0	52	2.7	-0.3	29	1.7	-02	-0.4	-02	1.4	19	1.6	0.3	01	0.9	-18	-1.3	-23	-1.8	-09	-1.9	0.6	-05	-2.2	-10	-0.9	-19	-2.5	-17	-1.3	-1.0	-09	-1.3	-18	-1.3
E_β	-5.1	-7.8	-78	-5.6	-48	-7.8	-54	-6.2	-78	-4.8	-5.3	-78	-7.8	-78	-7.8	-78	-7.8	-52	-7.8	-4.9	09	0.2	07	2.2	26	1.9	27	4.3	-0.2	19	2.7	06	0.4	13	1.8	20	2.0	0.7	01	0.6	-17	-2.2	-19	-1.6	-10	-1.1	-0.9	03	-2.7	-12	-1.7	-22	-2.3	-22	-0.9	-1.4	-20	-2.0	-22	-2.6
C_β	-7.8	-7.8	-44	-7.8	-78	-7.8	-78	-7.8	-10	-7.8	-7.8	-78	-7.8	-78	-3.1	-78	-3.9	-78	-7.8	-7.8	23	1.4	17	-0.5	03	0.5	-03	-0.2	6.1	07	0.5	11	1.4	19	1.1	17	1.6	1.0	11	0.5	-17	-2.6	-17	-4.0	-43	-2.1	-4.8	-48	2.8	-25	-2.5	-36	-4.5	-15	-1.3	-1.9	-22	-2.5	-09	-3.3
N_β	-5.9	-7.8	-50	-7.8	-78	-7.8	-47	-7.8	-78	-7.8	-7.8	-78	-5.9	-78	-7.8	-37	-7.8	-78	-7.8	-7.8	11	1.5	01	1.7	19	2.7	29	1.9	0.7	47	2.4	02	0.3	10	1.0	23	2.2	0.3	05	0.8	-11	-1.7	-13	-1.0	-10	-1.7	-1.1	-15	-1.4	06	-0.9	-19	-1.4	-21	-1.6	-0.5	-03	-4.1	-48	-2.3
Q_β	-6.1	-7.8	-78	-7.8	-78	-7.8	-78	-7.8	-78	-7.8	-7.8	-78	-6.1	-78	-7.8	-78	-7.8	-78	-7.8	-4.3	16	1.2	08	2.5	25	2.5	17	2.7	0.5	24	4.2	07	1.1	11	1.4	20	2.1	0.7	09	1.0	-14	-2.4	-22	-1.3	-16	-1.5	-1.7	-10	-3.2	-11	-0.1	-16	-2.9	-22	-1.4	-1.4	-15	-1.2	-27	-2.1
I_β	-7.8	-7.8	-69	-7.8	-60	-7.8	-78	-5.8	-78	-6.0	-7.8	-68	-7.8	-78	-5.6	-66	-6.4	-53	-4.9	-7.8	13	0.0	27	0.4	05	0.6	-02	0.6	1.1	02	0.7	33	2.6	19	0.1	03	1.1	1.8	09	1.3	-23	-3.8	-06	-3.1	-36	-3.0	-3.3	-34	-2.9	-29	-2.5	-01	-1.2	-21	-2.1	-3.6	-30	-1.9	-18	-2.5
L_β	-6.8	-6.1	-53	-5.8	-69	-7.8	-50	-7.4	-45	-5.9	-5.4	-78	-6.2	-55	-4.5	-78	-7.8	-78	-7.8	-7.8	12	-0.1	21	0.9	07	0.4	-04	0.4	1.4	03	1.1	26	3.4	27	0.7	06	0.9	2.1	12	1.4	-25	-3.9	-11	-4.5	-24	-2.3	-2.6	-41	-1.6	-31	-2.7	-11	-0.7	-16	-2.3	-3.0	-18	-1.4	-14	-1.5
M_β	-5.5	-7.8	-78	-3.4	-78	-7.8	-42	-7.8	-78	-7.8	-7.8	-78	-7.8	-78	-3.2	-78	-4.0	-40	-7.8	-7.8	20	0.5	20	0.9	07	1.1	-02	1.3	1.9	10	1.1	19	2.7	42	0.8	16	1.8	2.2	08	1.6	-31	-2.4	-32	-2.6	-44	-3.3	-3.4	-18	-4.8	-20	-2.1	-03	-0.9	00	-2.3	-3.2	-18	-1.6	-11	-1.1
P_β	-5.5	-7.8	-78	-7.8	-46	-3.0	-42	-7.8	-78	-3.6	-7.8	-78	-5.5	-78	-7.8	-78	-7.8	-78	-7.8	-3.7	15	0.4	10	0.5	15	1.3	14	1.8	1.1	10	1.4	01	0.7	08	6.1	15	1.1	-0.6	-02	0.2	-11	-2.0	-08	-1.5	-06	-0.9	-0.6	-12	-1.6	-14	-1.1	-28	-2.0	-06	1.0	-2.0	-12	-1.3	-48	-3.5
S_β	-7.8	-5.2	-78	-7.8	-78	-4.4	-56	-7.8	-78	-4.0	-7.8	-78	-5.8	-78	-7.8	-40	-7.8	-78	-7.8	-5.1	22	1.9	10	1.7	20	1.8	19	2.0	1.7	23	2.0	03	0.6	16	1.5	36	2.6	0.8	06	1.0	-17	-1.3	-09	-1.5	-10	-2.0	-1.3	-13	-1.6	-15	-0.9	-36	-2.1	-20	-2.0	-0.3	-07	-2.4	-24	-2.8
T_β	-7.8	-5.5	-78	-7.8	-78	-7.8	-44	-7.8	-78	-4.4	-3.9	-78	-5.6	-78	-7.8	-60	-4.8	-78	-7.8	-5.4	17	0.9	14	1.7	20	1.3	16	2.0	1.6	22	2.1	11	0.9	18	1.1	26	3.6	0.8	07	0.7	-14	-2.3	-10	-1.1	-16	-2.0	-1.9	-13	-1.7	-20	-2.4	-17	-2.4	-18	-1.8	-0.8	-04	-1.5	-12	-2.4
F_β	-7.0	-7.8	-78	-7.8	-62	-4.5	-48	-6.6	-78	-5.2	-4.7	-44	-6.0	-78	-4.8	-78	-7.8	-45	-4.1	-7.8	11	0.3	15	0.8	02	1.5	03	0.7	1.0	03	0.7	18	2.1	22	-0.6	08	0.8	4.1	28	3.0	-31	-2.9	-20	-1.9	-21	-1.1	-3.0	-25	-4.1	-23	-3.6	-20	-1.1	-18	-2.0	-2.8	-26	-0.2	-08	-0.8
W_β	-7.8	-7.8	-44	-7.8	-78	-7.8	-78	-7.8	-78	-7.8	-7.8	-43	-5.3	-78	-7.8	-78	-2.9	-39	-2.4	-7.8	09	0.3	09	0.3	09	1.7	01	0.1	1.1	05	0.9	09	1.2	08	-0.2	06	0.7	2.8	62	2.9	-25	-2.6	-17	-4.0	-23	-2.1	-3.8	-26	-4.8	-25	-1.2	-16	-2.1	-25	-2.0	-2.7	-29	-0.9	24	-0.7
Y_β	-5.8	-7.8	-78	-7.8	-78	-4.3	-78	-7.8	-78	-7.8	-7.8	-78	-5.8	-78	-4.5	-55	-5.3	-78	-7.8	-5.0	12	0.8	11	1.1	11	2.0	09	0.6	0.5	08	1.0	13	1.4	16	0.2	10	0.7	3.0	29	4.5	-21	-2.8	-20	-2.4	-29	-1.1	-2.5	-31	-1.5	-20	-1.7	-16	-2.8	-39	-1.7	-2.2	-20	-0.4	-06	0.6
A_c	-1.1	-1.2	-15	-1.5	-17	-1.8	-12	-1.8	-29	-1.2	-1.4	-28	-1.7	-15	-0.8	-09	-1.4	-29	-1.9	-2.2	-07	-1.3	-20	-1.7	-14	-3.2	-18	-1.7	-1.7	-11	-1.4	-23	-2.5	-31	-1.1	-17	-1.4	-3.1	-25	-2.1	27	0.7	13	0.4	08	0.2	0.6	09	1.1	05	1.0	05	0.6	13	1.0	1.3	10	0.5	-01	0.7
G_c	-2.3	-0.4	-35	-2.5	-21	-1.9	-20	-2.0	-26	-2.2	-2.1	-35	-3.0	-45	-1.5	-17	-2.4	-26	-3.3	-3.1	-16	0.3	-32	-2.4	-23	-2.7	-13	-2.2	-2.6	-17	-2.4	-38	-3.9	-24	-2.0	-13	-2.3	-2.9	-26	-2.8	07	3.2	-03	0.2	04	0.5	0.6	06	-0.6	10	0.6	-06	-0.8	-01	0.0	0.6	02	-0.3	-03	-0.5
V_c	-1.6	-2.2	-08	-2.7	-24	-1.7	-31	-2.5	-30	-2.3	-3.4	-15	-1.9	-16	-1.3	-19	-1.5	-19	-1.2	-1.5	-10	-1.7	-08	-1.7	-16	-2.3	-23	-1.9	-1.7	-13	-2.2	-06	-1.1	-32	-0.8	-09	-1.0	-2.0	-17	-2.0	13	-0.3	32	0.6	03	0.3	-0.4	03	1.1	-01	0.2	28	1.7	15	0.7	0.3	12	1.3	01	0.7
R_c	-2.0	-1.4	-31	-0.6	-08	-1.2	-17	-2.5	-20	-1.0	-1.0	-43	-2.5	-19	-1.2	-13	-2.1	-25	-1.6	-2.9	-25	-1.4	-23	0.4	-07	-1.7	-18	-1.6	-4.0	-10	-1.3	-31	-4.5	-26	-1.5	-15	-1.1	-1.9	-40	-2.4	04	0.2	06	3.6	20	1.2	0.4	12	-0.5	10	1.6	02	0.3	08	0.4	0.8	06	0.1	01	0.5
K_c	-2.1	-2.3	-27	-0.7	-06	-1.9	-14	-1.9	-27	-0.8	-1.3	-29	-2.4	-19	-1.0	-11	-1.6	-24	-2.7	-2.8	-22	-1.9	-30	-0.8	01	-1.6	-09	-1.0	-4.3	-10	-1.6	-36	-2.4	-44	-0.6	-10	-1.6	-2.1	-23	-2.9	08	0.4	03	2.0	29	0.8	1.0	14	-0.6	12	1.4	00	0.3	08	0.8	0.9	09	-0.6	-02	0.3
H_c	-1.9	-1.7	-32	-2.0	-24	0.9	-10	-1.8	-31	-1.7	-1.7	-21	-2.1	-21	-2.1	-18	-2.0	-19	-0.9	-1.6	-21	-0.6	-23	-2.1	-16	1.1	-19	-1.1	-2.1	-17	-1.5	-30	-2.3	-33	-0.9	-20	-2.0	-1.1	-21	-1.1	02	0.5	03	1.2	08	4.5	0.7	04	0.9	10	1.3	-01	0.0	07	0.3	0.9	05	0.7	07	1.7
D_c	-2.3	-1.7	-32	-2.2	-21	-2.0	-05	-1.2	-38	-1.4	-2.3	-43	-2.9	-35	-1.8	-16	-2.7	-24	-2.6	-1.7	-27	-1.7	-31	-2.0	-18	-2.5	06	-0.9	-4.8	-11	-1.7	-33	-2.6	-34	-0.6	-13	-1.9	-3.0	-38	-2.5	06	0.6	-04	0.4	10	0.7	3.2	16	-0.5	16	1.0	-06	-0.4	-03	0.5	1.1	09	-0.5	-05	0.2
E_c	-2.2	-1.7	-35	-1.6	-11	-2.5	-12	-0.4	-26	-1.1	-1.3	-34	-2.2	-19	-1.1	-11	-2.3	-25	-2.3	-1.7	-26	-1.7	-30	-0.6	-07	-0.8	-05	0.3	-4.8	-15	-1.0	-34	-4.1	-18	-1.2	-13	-1.3	-2.5	-26	-3.1	09	0.6	03	1.2	14	0.4	1.6	29	-1.0	10	1.8	02	-0.1	06	0.8	0.9	07	-0.3	-07	0.1
C_c	-1.9	-1.4	-25	-3.7	-23	-3.2	-29	-3.7	26	-2.3	-2.8	-21	-2.1	-48	-4.8	-21	-1.5	-22	-1.2	-1.9	-27	-1.6	-23	-2.7	-17	-1.1	-22	-2.7	2.8	-14	-3.2	-29	-1.6	-48	-1.6	-16	-1.7	-4.1	-48	-1.5	11	-0.6	11	-0.5	-06	0.9	-0.5	-10	6.1	02	-0.3	04	0.3	-05	-0.1	0.3	08	0.1	-07	0.1
N_c	-2.2	-1.8	-31	-2.0	-13	-1.8	-09	-1.7	-34	-0.3	-1.0	-27	-2.4	-20	-1.5	-11	-1.8	-27	-2.5	-2.1	-26	-1.6	-32	-1.6	-13	-2.2	-10	-1.2	-2.5	06	-1.1	-29	-3.1	-20	-1.4	-15	-2.0	-2.3	-25	-2.0	05	1.0	-01	1.0	12	1.0	1.6	10	0.2	30	1.1	-02	-0.2	02	0.1	1.2	11	0.2	02	0.5
Q_c	-2.2	-2.1	-17	-1.4	-15	-1.5	-13	-1.4	-35	-0.8	-0.7	-23	-2.4	-30	-1.1	-13	-1.4	-38	-2.3	-1.2	-19	-2.5	-29	-1.8	-10	-1.2	-09	-1.7	-2.5	-09	-0.1	-25	-2.7	-21	-1.1	-09	-2.4	-3.6	-12	-1.7	10	0.6	02	1.6	14	1.3	1.0	18	-0.3	11	3.1	03	0.3	11	0.6	1.0	10	0.1	05	0.6
I_c	-2.1	-1.4	-10	-2.1	-28	-1.0	-20	-2.9	-36	-3.1	-2.1	-10	-1.2	-15	-1.5	-23	-2.3	-15	-2.0	-1.3	-23	-2.3	-12	-1.5	-34	-1.9	-19	-2.2	-3.6	-19	-1.6	-01	-1.1	-03	-2.8	-36	-1.7	-2.0	-16	-1.6	05	-0.6	28	0.2	00	-0.1	-0.6	02	0.4	-02	0.3	35	2.4	19	0.5	-0.1	07	1.7	09	1.0
L_c	-1.8	-1.9	-11	-2.2	-19	-2.5	-22	-2.6	-19	-2.2	-1.9	-09	-0.2	-21	-2.2	-17	-1.9	-07	-1.7	-1.1	-22	-2.7	-13	-2.0	-20	-1.9	-25	-2.3	-4.5	-14	-2.9	-12	-0.7	-09	-2.0	-21	-2.4	-1.1	-21	-2.8	06	-0.8	17	0.3	03	0.0	-0.4	-01	0.3	-02	0.3	24	3.3	23	0.2	0.0	06	1.8	13	1.2
M_c	-1.6	-2.5	-17	-2.7	-23	-4.8	-19	-3.0	-48	-2.9	-1.4	-07	-0.7	02	-1.9	-22	-2.7	-15	-0.5	-1.4	-33	-2.0	-18	-2.8	-22	-2.7	-17	-2.2	-1.5	-21	-2.2	-21	-1.6	00	-0.6	-20	-1.6	-1.8	-25	-3.9	13	-0.1	15	0.8	08	0.7	-0.3	06	-0.5	02	1.1	19	2.3	42	0.1	0.4	08	1.5	02	1.3
P_c	-2.4	-1.6	-31	-2.8	-22	-2.6	-19	-2.3	-48	-2.8	-2.4	-34	-3.1	-32	0.6	-19	-2.5	-40	-2.6	-2.4	-16	-2.1	-21	-1.4	-14	-1.7	-13	-0.9	-1.3	-16	-1.4	-21	-2.3	-23	1.0	-20	-1.8	-2.0	-20	-1.7	10	0.0	07	0.4	08	0.3	0.5	08	-0.1	01	0.6	05	0.2	01	3.6	0.8	04	0.3	-05	0.2
S_c	-1.9	-1.3	-27	-1.9	-15	-2.7	-19	-1.5	-21	-1.7	-2.2	-40	-2.7	-20	-0.8	-05	-1.2	-33	-2.5	-2.3	-17	-1.4	-26	-2.2	-14	-1.8	-10	-1.4	-1.9	-05	-1.4	-36	-3.0	-32	-2.0	-03	-0.8	-2.8	-27	-2.2	13	0.6	03	0.8	09	0.9	1.1	09	0.3	12	1.0	-01	0.0	04	0.8	2.7	17	0.0	04	0.3
T_c	-2.1	-2.1	-22	-2.1	-17	-2.0	-20	-2.1	-29	-2.0	-2.0	-24	-2.7	-18	-1.8	-14	-0.7	-28	-3.3	-2.1	-16	-2.3	-20	-1.0	-09	-1.3	-09	-2.0	-2.2	-03	-1.5	-30	-1.8	-18	-1.2	-07	-0.4	-2.6	-29	-2.0	10	0.2	12	0.6	09	0.5	0.9	07	0.8	11	1.0	07	0.6	08	0.4	1.7	29	0.3	03	0.2
F_c	-2.1	-1.9	-18	-2.4	-22	-1.5	-21	-2.4	-48	-1.6	-2.6	-18	-0.8	-09	-1.9	-19	-2.0	04	-0.9	-0.4	-20	-3.4	-27	-1.5	-25	-1.8	-13	-2.0	-2.5	-41	-1.2	-19	-1.4	-16	-1.3	-24	-1.5	-0.2	-09	-0.4	05	-0.3	13	0.1	-06	0.7	-0.5	-03	0.1	02	0.1	17	1.8	15	0.3	0.0	03	4.0	23	2.9
W_c	-3.6	-2.0	-20	-1.8	-44	-2.7	-17	-2.8	-19	-3.4	-1.9	-12	-1.6	-06	-2.0	-30	-1.8	-12	1.9	0.0	-38	-3.4	-18	-1.6	-13	-4.8	-18	-2.2	-0.9	-48	-2.7	-18	-1.4	-11	-4.8	-24	-1.2	-0.8	24	-0.6	-01	-0.3	01	0.1	-02	0.7	-0.5	-07	-0.7	02	0.5	09	1.3	02	-0.5	0.4	03	2.3	60	2.4
Y_c	-2.3	-2.3	-29	-2.2	-24	-1.3	-22	-2.8	-48	-1.0	-2.7	-28	-1.9	-15	-1.8	-14	-1.6	-09	-0.7	0.5	-20	-2.2	-27	-1.8	-16	-1.3	-13	-2.6	-3.3	-23	-2.1	-25	-1.5	-11	-3.5	-28	-2.4	-0.8	-07	0.6	07	-0.5	07	0.5	03	1.7	0.2	01	0.1	05	0.6	10	1.2	13	0.2	0.3	02	2.9	24	4.3
