# Supplementary material for: (PS)2-v2: template-based protein structure prediction server
Source: BMC Bioinformatics. 2009 Oct 31;10:366. doi: 10.1186/1471-2105-10-366 (PMC2775752; doi:10.1186/1471-2105-10-366)
Supplement: Additional file 3 — Table S1. The summary of 154 TBM targets in CASP8. [file 1471-2105-10-366-S3.pdf]

Table S1. **The summary of 154 TBM targets in CASP8**

Target	Residues	Best templates	LGA_S (%)	RMSD	Sequence identity (%)
T0388-D1	164	2p31A	92.16	0.93	56.5
T0389-D1	134	2oucA	83.87	1.96	30.7
T0390-D1	124	1shxB	93.59	1.36	52.1
T0391-D1	128	1z01_F	72.34	1.89	18.3
T0392-D1	82	1vaeA	93.88	1.56	84.2
T0393-D1	159	2f1kA	84.24	2.00	18.3
T0393-D2	99	2i76A	89.17	1.97	24.2
T0394-D1	238	1h2eA	74.08	1.67	26.1
T0395-D1	218	1surA	57.48	2.03	17.5
T0396-D1	102	1jraA	93.11	1.33	24.2
T0397-D2	68	2yu9B	89.00	1.99	8.8
T0398-D1	143	2rirG	99.12	0.67	58.0
T0398-D2	147	2rirE	99.57	0.63	66.0
T0399-D1	162	1s0wC	54.95	2.59	16.7
T0400-D1	155	2q7bA	94.01	1.32	33.3
T0401-D1	127	2prvB	67.58	2.18	21.1
T0402-D1	114	1rfeA	91.20	1.62	18.8
T0404-D1	79	2cz4C	96.18	0.98	28.2
T0406-D1	147	2ou6A	69.82	1.96	13.3
T0407-D1	231	2anuC	57.38	2.27	22.8
T0407-D2	97	1z9oA	59.11	2.38	10.0
T0408-D1	98	2af7F	85.74	2.12	19.6
T0409-D1	62	2vnuD	83.76	1.55	12.7
T0411-D1	118	1e0cA	80.10	1.85	22.3
T0412-D1	165	2o0yB	83.71	1.66	18.2
T0413-D1	282	1jjiD	44.05	2.40	15.3
T0414-D1	127	2b5hA	71.97	1.96	7.8
T0415-D1	107	3bs1A	78.21	1.79	21.5
T0416-D1	232	2qgnA	93.82	1.39	46.8
T0417-D1	153	2cnmC	77.10	2.00	14.7
T0418-D1	141	2hszB	91.88	1.46	26.1
T0418-D2	69	2ah5A	90.49	1.38	24.2
T0419-D1	224	1e98A	47.09	2.42	14.7
T0419-D2	241	2plrA	45.58	2.44	12.1
T0420-D1	168	1hv9B	81.14	1.94	17.3
T0421-D1	221	2plrA	47.12	2.29	19.8
T0422-D1	200	3b9pA	85.21	1.40	57.2
T0422-D2	80	2qpaB	92.77	1.53	21.5
T0423-D1	147	2otmC	93.38	1.13	32.4
T0424-D1	175	3cddD	88.63	1.67	21.4
T0424-D2	84	1wruA	88.60	1.81	25.6
T0424-D3	57	3cddF	90.69	1.66	19.6
T0425-D1	179	1jwqA	83.03	1.97	24.3
T0426-D1	257	2fw4A	98.82	0.77	60.3
T0427-D1	218	1ppjA	74.81	1.93	14.4
T0427-D2	184	1hr7C	80.35	1.96	15.7
T0428-D1	229	1xq9B	98.72	0.78	63.8
T0429-D1	55	2f5kB	92.93	1.23	22.6
T0429-D2	75	2d9tA	68.77	1.80	10.2
T0430-D1	138	1zwsB	55.73	1.88	22.4
T0430-D2	189	2bheA	50.17	2.05	22.9
T0431-D1	101	2iagB	86.97	1.53	32.3
T0431-D2	357	2iagA	88.74	1.56	33.1
T0432-D1	130	2dkwA	86.85	1.59	66.9
T0433-D1	199	1ybvA	91.92	1.65	21.6
T0434-D1	151	1mvhA	63.75	2.17	23.4
T0435-D1	118	2qpwA	85.60	1.57	32.4
T0436-D1	405	1vp4B	59.20	2.23	19.2

T0437-D1	68	2jz5A	91.26	1.82	33.9
T0438-D1	164	2oasA	90.65	1.44	29.8
T0438-D2	223	2oasA	97.21	1.06	49.8
T0440-D1	275	2yz5A	79.69	1.64	26.6
T0441-D1	124	2driA	89.00	1.78	17.2
T0441-D2	146	1sxgA	93.02	1.24	24.7
T0442-D1	157	2pifB	92.19	1.51	79.4
T0442-D2	73	2pifB	92.19	1.51	69.9
T0443-D1	66	2aplA	68.10	2.54	11.9
T0443-D3	66	1onvA	68.03	1.74	7.0
T0444-D1	276	1jk0A	98.00	0.87	66.3
T0445-D1	155	2hf2B	93.92	1.32	29.4
T0445-D2	107	1nf2C	85.13	1.99	9.7
T0446-D1	57	3b77F	86.67	1.95	18.2
T0446-D2	50	2j0kA	91.23	1.72	16.3
T0447-D1	542	1eg7A	95.15	1.12	52.3
T0448-D1	207	3bzwE	89.15	1.57	26.0
T0449-D1	296	2cisA	62.10	1.80	21.5
T0450-D1	491	2rghA	91.31	1.41	42.2
T0451-D1	127	1s5aD	84.14	1.87	20.8
T0452-D1	156	3c8mA	89.16	1.83	29.6
T0452-D2	163	2ejwA	93.68	1.30	42.5
T0453-D1	86	2plsI	91.42	1.66	38.8
T0454-D1	52	2jj7A	97.76	0.98	36.5
T0454-D2	140	1vi0B	75.11	2.16	10.2
T0455-D1	139	3bwgB	93.16	1.59	25.2
T0456-D1	87	2gs7B	88.75	1.67	22.6
T0456-D2	175	2qg5B	90.02	1.20	44.4
T0457-D1	194	1wpmB	63.49	2.30	16.7
T0457-D2	118	1ir6A	59.63	2.55	12.5
T0458-D1	77	2okaA	99.39	0.71	55.8
T0459-D1	91	1yyvA	91.67	1.56	25.8
T0460-D1	80	2fug5	56.59	2.51	16.7
T0461-D1	154	2b3jB	89.90	1.32	31.5
T0462-D1	70	2gcxA	82.80	2.05	33.3
T0462-D2	63	2gcxA	76.93	2.17	17.2
T0463-D1	211	1he3A	81.59	1.94	23.3
T0464-D1	69	1b6bA	50.32	2.62	5.6
T0466-D1	72	1xjvA	74.89	1.94	1.6
T0468-D1	61	2oq0C	66.59	2.54	11.5
T0469-D1	63	2fi0A	79.54	2.17	23.0
T0470-D1	111	2qgsA	92.19	1.92	35.1
T0470-D2	77	2qgsB	96.54	1.19	49.3
T0471-D1	88	2r3sA	84.88	1.99	10.6
T0472-D1	58	1uwbA	88.70	1.99	15.5
T0472-D2	45	3bidE	90.99	1.34	18.6
T0473-D1	60	2fi0A	82.34	2.19	21.1
T0474-D1	41	2ay0C	97.88	0.94	12.2
T0475-D1	118	1qjgF	89.44	1.80	22.4
T0476-D1	87	2zbzA	46.85	2.84	12.5
T0477-D1	240	2db3A	82.43	1.81	32.6
T0478-D1	126	1xlyA	70.28	2.50	8.6
T0478-D2	130	1xlyB	68.69	2.29	9.5
T0479-D1	122	1zkiB	95.23	1.21	29.2
T0480-D1	30	1dl6A	89.62	1.74	23.3
T0481-D1	135	1rxqA	67.20	2.21	14.5
T0483-D1	273	1xr1A	84.10	1.81	31.5
T0485-D1	207	2o57C	70.41	2.19	20.6
T0486-D1	214	2vssF	92.29	1.55	24.8
T0487-D1	194	2f8sB	62.09	2.14	18.8
T0487-D2	134	2f8sB	49.37	2.71	13.6
T0487-D3	71	2f8sB	67.56	2.22	7.6

T0487-D4	87	1si2A	72.02	2.34	12.2
T0487-D5	147	1w9hA	54.94	2.65	14.7
T0488-D1	86	2qg1A	95.98	1.08	35.3
T0489-D1	210	1zxvA	38.36	2.70	10.4
T0490-D1	361	1ryiA	72.44	2.04	16.8
T0491-D1	96	1wocD	92.40	1.49	27.7
T0492-D1	69	2gcxA	81.58	2.00	24.6
T0493-D1	149	2is4A	89.58	1.85	23.5
T0494-D1	345	2eu9A	82.74	1.56	32.7
T0495-D1	139	2okfB	46.66	2.47	11.2
T0496-D2	45	3bvoB	99.13	0.78	13.3
T0497-D1	124	2qeaA	88.52	2.00	29.8
T0498-D1	45	2fs1A	94.64	1.22	62.2
T0499-D1	56	1qkzA	93.48	1.40	60.7
T0501-D1	213	1wpnB	57.16	2.29	17.0
T0501-D2	126	1ir6A	62.17	2.36	11.9
T0502-D1	93	2k50A	87.64	1.68	27.5
T0503-D1	144	1o3rA	75.86	2.06	16.7
T0504-D1	62	2f5kA	86.16	1.27	7.3
T0504-D2	90	2hqeA	62.17	2.11	14.3
T0504-D3	53	2r5aA	95.44	1.42	17.0
T0505-D1	159	1rkqA	92.85	1.29	35.7
T0505-D2	104	2hf2B	68.76	1.96	5.5
T0506-D1	137	2arzA	92.07	1.48	23.1
T0506-D2	78	2arzA	88.29	2.10	22.1
T0507-D1	124	2pidB	70.71	2.12	9.5
T0508-D1	174	1eizA	91.97	1.42	32.0
T0509-D1	209	2hnkB	86.77	1.88	20.5
T0510-D1	151	1r2zA	50.37	2.04	4.7
T0510-D2	68	2f5sA	73.33	2.29	18.2
T0511-D1	249	1vl8B	82.09	1.70	25.0
T0512-D1	320	1l0qB	65.73	2.12	18.8
T0513-D1	208	2fkbA	64.85	2.06	14.7
T0514-D1	144	2zf8A	47.10	2.70	8.0
