# Supplementary material for: (PS)2-v2: template-based protein structure prediction server
Source: BMC Bioinformatics. 2009 Oct 31;10:366. doi: 10.1186/1471-2105-10-366 (PMC2775752; doi:10.1186/1471-2105-10-366)
Supplement: Additional file 4 — Table S2. The GDT_TS scores of the (PS)2-original, (PS)2-CASP8 and (PS)2-v2 servers on 154 TBM targets. [file 1471-2105-10-366-S4.pdf]

Table S2. The GDT_TS scores of the (PS)^2-original, (PS)^2-CASP8 and (PS)^2-v2 servers on 154 TBM targets

CASP8 targets	(PS)^2-original [1]		(PS)^2-CASP8 [2]		(PS)^2-v2 (This study)			Different GDT_TS between (PS)^2-CASP8 and (PS)^2-v2
	Templates	GDT_TS	Templates	GDT_TS	Templates	w^{s2a2}	GDT_TS	
T0388-D1	2p31A	90.5	2p31A	90.6	2p31A	0.64	90.6	0
T0389-D1	1hzmA	36.6	2oucA	67.2	2oucA	0.64	67.2	0
T0390-D1	1shxA	90.5	1shxA	90.3	1shxA	0.64	90.3	0
T0391-D1	1z01A	61.5	2e4qA	57.4	2e4qA	0.64	57.4	0
T0392-D1	1vaeA	87.5	1vaeA	87.5	1vaeA	0.64	87.5	0
T0393-D1	1teaA	63.2	2h78A	59.3	2h78A	0.64	59.3	0
T0393-D2	1teaA	35.1	2h78A	31.6	2h78A	0.64	31.6	0
T0394-D1	1xq9A	51.1	1xq9A	51.7	1xq9A	0.64	51.7	0
T0395-D1	2oq2C	36.4	1zunA	49.1	1zunA	0.64	49.1	0
T0396-D1	1oqcA	86.0	1oqcA	86.0	1oqcA	0.64	86.0	0
T0397-D2	1w9iA	18.0	1wltA	26.1	1wltA	0.64	26.1	0
T0398-D1	2rirE	94.1	2rirE	94.1	2rirE	0.64	94.1	0
T0398-D2	2rirE	98.6	2rirE	98.6	2rirE	0.64	98.6	0
T0399-D1	2g2uB	40.4	2g2uB	42.6	2g2uB	0.64	42.6	0
T0400-D1	2q7bA	85.2	2q7bA	90.5	2q7bA	0.64	90.5	0
T0401-D1	2icgA	54.5	2prvA	61.8	2prvA	0.64	61.8	0
T0402-D1	2i02A	82.2	2i02A	81.4	2i02A	0.64	81.4	0
T0404-D1	2cz4A	93.0	2cz4A	92.7	2cz4A	0.64	92.7	0
T0406-D1	2f22B	51.0	2hkvA	62.9	2hkvA	0.64	62.9	0
T0407-D1	2hnhA	34.1	2anuA	62.6	2anuA	0.64	62.6	0
T0407-D2 [b]	2hnhA	16.5	2anuA	0	2iakB	0.64	17.8	17.8
T0408-D1 [c]	2af7D	79.8	1vkeE	63.5	2af7D	0.64	75.3	11.8
T0409-D1 [c]	1wv3A	29.4	1bkbA	75.4	1bkbA	0.4	77.8	2.4
T0411-D1	1uarA	66.1	2hhgA	74.4	2hhgA	0.64	74.4	0
T0412-D1	1mkmB	61.2	2o99C	73.3	2o99C	0.64	73.3	0
T0413-D1	2ecfA	32.9	1jjfA	35.7	1jjfA	0.64	35.7	0
T0414-D1	1fi2A	44.7	2pa7A	50.0	2pa7A	0.64	50.0	0
T0415-D1 [c]	3bs1A	72.9	3bs1A	74.5	3bs1A	0.4	75.0	0.5
T0416-D1	2qgnA	69.0	2qgnA	86.0	2qgnA	0.64	86.0	0
T0417-D1	2j8mA	55.4	2cntA	65.9	2cntA	0.64	65.9	0
T0418-D1 [c]	2nyvA	85.6	2pibA	82.3	2hi0A	0.64	86.0	3.7
T0418-D2 [c]	2nyvA	76.1	2pibA	69.6	2hi0A	0.64	72.8	3.2
T0419-D1	4tmkA	26.5	2jaqA	38.1	2jaqA	0.64	38.1	0
T0419-D2 [b]	4tmkA	5.0	2jaqA	0	2jaqA	0.64	36.6	36.6
T0420-D1 [c]	2v0hA	53.6	1e5kA	56.8	1fxjA	0.64	66.4	9.6
T0421-D1	4tmkA	25.0	2jaqA	40.4	2jaqA	0.64	40.4	0
T0422-D1	3b9pA	79.6	3b9pA	78.3	3b9pA	0.64	78.3	0
T0422-D2	3b9pA	86.9	3b9pA	86.9	3b9pA	0.64	86.9	0
T0423-D1	2otmB	91.5	2otmB	91.2	2otmB	0.64	91.2	0
T0424-D1	1wruA	76.1	3cddD	78.0	3cddD	0.64	78.0	0
T0424-D2	1wruA	75.9	3cddD	78.6	3cddD	0.64	78.6	0
T0424-D3	1wruA	78.5	3cddD	79.8	3cddD	0.64	79.8	0
T0425-D1	1jwqA	60.3	1jwqA	64.0	1jwqA	0.64	64.0	0
T0426-D1	1lugA	94.8	2nmxA	96.4	2nmxA	0.64	96.4	0
T0427-D1	1kb9A	54.7	1hr6D	69.5	1hr6D	0.64	69.5	0
T0427-D2	1kb9A	56.9	1hr6D	63.2	1hr6D	0.64	63.2	0
T0428-D1	1xq9A	95.6	1xq9A	95.6	1xq9A	0.64	95.6	0
T0429-D1	2hfvA	15.0	1oh4A	23.6	1oh4A	0.64	23.6	0
T0429-D2	2hfvA	21.3	1oh4A	28.3	1oh4A	0.64	28.3	0
T0430-D1 [a]	1unlA	38.2	1unlA	27.7	2r3iA	0.64	36.4	8.7
T0430-D2 [a]	1unlA	35.3	1unlA	41.1	2r3iA	0.64	35.1	-6
T0431-D1	3b6hB	79.0	3b6hB	73.0	3b6hB	0.64	73.0	0
T0431-D2	3b6hB	80.0	3b6hB	75.2	3b6hB	0.64	75.2	0
T0432-D1	2dkwA	81.3	2dkwA	81.0	2dkwA	0.64	81.0	0

T0433-D1	1q7bA	69.7	1q7bA	76.8	1q7bA	0.64	76.8	0
T0434-D1 [a]	2o8jB	52.2	2rfiA	58.4	2qpwA	0.64	63.4	5
T0435-D1	2qpwA	80.3	2qpwA	79.7	2qpwA	0.64	79.7	0
T0436-D1	1x0mA	60.4	2dtvA	59.3	2dtvA	0.64	59.3	0
T0437-D1	2jz5A	84.6	2jz5A	84.9	2jz5A	0.64	84.9	0
T0438-D1	2oasA	86.0	2oasA	87.8	2oasA	0.64	87.8	0
T0438-D2	2oasA	93.0	2oasA	92.7	2oasA	0.64	92.7	0
T0440-D1	2yxoB	69.5	2yxoB	71.6	2yxoB	0.64	71.6	0
T0441-D1	1jftA	73.6	1jftA	73.4	1jftA	0.64	73.4	0
T0441-D2	1jftA	80.8	1jftA	80.5	1jftA	0.64	80.5	0
T0442-D1	2pifB	93.8	2pifB	93.8	2pifB	0.64	93.8	0
T0442-D2	2pifB	95.9	2pifB	95.9	2pifB	0.64	95.9	0
T0443-D1	1ps1A	36.4	1tffA	33.0	1tffA	0.64	33.0	0
T0443-D3 [b]	1ps1A	30.3	1tffA	0	1p0zA	0.64	42.4	42.4
T0444-D1	1h0oA	88.1	1h0oA	87.5	1h0oA	0.64	87.5	0
T0445-D1	2hf2B	85.8	2hf2B	87.9	2hf2B	0.64	87.9	0
T0445-D2	2hf2B	62.2	2hf2B	66.6	2hf2B	0.64	66.6	0
T0446-D1	3b77A	67.5	3b77A	71.9	3b77A	0.64	71.9	0
T0446-D2	3b77A	68.5	3b77A	67.0	3b77A	0.64	67.0	0
T0447-D1	1eg7A	88.8	1eg7A	88.6	1eg7A	0.64	88.6	0
T0448-D1	3bzwA	75.4	3bzwA	76.2	3bzwA	0.64	76.2	0
T0449-D1 [a,c]	1nsxB	42.6	1lurA	54.5	1snzA	0.8	55.2	0.7
T0450-D1	2rghA	84.0	2rghA	79.3	2rghA	0.64	79.3	0
T0451-D1 [c]	1s5aB	65.2	1s5aB	67.1	1tuhA	0.64	68.9	1.8
T0452-D1	2ejwA	65.2	2ejwA	77.7	2ejwA	0.64	77.7	0
T0452-D2	2ejwA	88.3	2ejwA	89.6	2ejwA	0.64	89.6	0
T0453-D1	2plsA	83.4	2plsA	83.4	2plsA	0.64	83.4	0
T0454-D1	2iaiA	82.2	1pb6A	91.8	1pb6A	0.64	91.8	0
T0454-D2	2iaiA	64.1	1pb6A	59.3	1pb6A	0.64	59.3	0
T0455-D1	2ooiB	78.6	3bwgB	86.3	3bwgB	0.64	86.3	0
T0456-D1	2qg5A	75.9	2qg5A	75.6	2qg5A	0.64	75.6	0
T0456-D2	2qg5A	78.7	2qg5A	79.4	2qg5A	0.64	79.4	0
T0457-D1 [c]	1ir6A	41.9	1k20A	58.0	1k20A	0.8	60.6	2.6
T0457-D2 [c]	1ir6A	37.9	1k20A	37.5	1k20A	0.8	45.8	8.3
T0458-D1	2okaA	96.1	2fa8B	95.8	2fa8B	0.64	95.8	0
T0459-D1	2f2eA	75.3	1z7uA	76.7	1z7uA	0.64	76.7	0
T0460-D1 [a]	2h2wA	-	2pr5A	23.1	1q33A	0.64	32.2	9.1
T0461-D1	2nx8A	83.1	2nx8A	84.7	2nx8A	0.64	84.7	0
T0462-D1	2gcxA	72.1	2gcxA	71.1	2gcxA	0.64	71.1	0
T0462-D2	2gcxA	75.3	2gcxA	77.4	2gcxA	0.64	77.4	0
T0463-D1 [c]	2c29F	60.3	1r6dA	56.9	1r6dA	0.4	58.6	1.7
T0464-D1	2jnyA	42.0	2pk7A	46.4	2pk7A	0.64	46.4	0
T0466-D1	1ogoX	21.2	1xq4A	32.3	1xq4A	0.64	32.3	0
T0468-D1 [c]	1gsoA	27.0	1bbpA	26.6	1bbpA	0.4	28.3	1.7
T0469-D1	2fi0A	75.8	2fi0A	75.8	2fi0A	0.64	75.8	0
T0470-D1	2pjqB	79.3	2qgsA	85.6	2qgsA	0.64	85.6	0
T0470-D2	2pjqB	87.7	2qgsA	94.5	2qgsA	0.64	94.5	0
T0471-D1 [c]	2absA	24.4	2nwrA	32.7	1nv8A	0.64	61.7	29
T0472-D1	3bidE	73.7	3bidE	74.6	3bidE	0.64	74.6	0
T0472-D2	3bidE	86.7	3bidE	86.7	3bidE	0.64	86.7	0
T0473-D1 [c]	2fi0A	75.8	2fi0A	75.4	2fi0A	0.8	74.2	-1.2
T0474-D1 [a]	1vr6A	37.2	1fadA	51.2	2cpgB	0.64	78.7	27.5
T0475-D1 [c]	1ohpA	75.4	1ohpA	75.6	1ohpA	0.4	79.2	3.6
T0476-D1 [c]	2gx9A	23.3	2c5kT	22.7	2c5kT	0.8	23.9	1.2
T0477-D1 [c]	2db3A	75.1	2db3A	76.5	2db3A	0.4	76.0	-0.5
T0478-D1 [a,c]	1toaA	26.8	2v0oA	35.1	1h6gA	0.8	42.1	7
T0478-D2 [a,c]	1toaA	9.6	2v0oA	29.8	1h6gA	0.8	38.1	8.3
T0479-D1 [c]	1zkiA	89.1	1zkiA	88.9	1zkiA	0.4	90.4	1.5
T0480-D1	3cngC	60.8	2zkr2	59.2	2zkr2	0.64	59.2	0
T0481-D1	2f22B	64.1	2hkvA	64.1	2hkvA	0.64	64.1	0
T0483-D1	2qnjB	64.1	2qnjB	62.7	2qnjB	0.64	62.7	0
T0485-D1	3busA	59.3	1im8A	58.9	1im8A	0.64	58.9	0

T0486-D1	2hw5A	80.0	2pbpA	80.4	2pbpA	0.64	80.4	0
T0487-D1	1yvuA	59.7	2f8sA	62.9	2f8sA	0.64	62.9	0
T0487-D2	1yvuA	45.3	2f8sA	35.3	2f8sA	0.64	35.3	0
T0487-D3	1yvuA	47.5	2f8sA	65.1	2f8sA	0.64	65.1	0
T0487-D4	1yvuA	50.3	2f8sA	37.4	2f8sA	0.64	37.4	0
T0487-D5	1yvuA	51.0	2f8sA	51.9	2f8sA	0.64	51.9	0
T0488-D1	2he2A	84.3	2qg1A	92.7	2qg1A	0.64	92.7	0
T0489-D1	1ei9A	11.3	1jq5A	13.6	1jq5A	0.64	13.6	0
T0490-D1	2qcuB	60.6	1y56B	68.8	1y56B	0.64	68.8	0
T0491-D1 [c]	1wocC	82.6	1wocC	81.8	1wocC	0.4	82.8	1
T0492-D1	2gcxA	71.0	2h3jA	71.7	2h3jA	0.64	71.7	0
T0493-D1	2is6A	36.2	1pjrA	74.0	1pjrA	0.64	74.0	0
T0494-D1	2eu9A	74.1	2eu9A	72.5	2eu9A	0.64	72.5	0
T0495-D1	1y0kA	11.2	1gefA	37.6	1gefA	0.64	37.6	0
T0496-D2 [c]	1pp9A	55.0	2pbrA	37.2	1dd5A	0.64	71.1	33.9
T0497-D1	2qeaA	75.2	2i02A	74.6	2i02A	0.64	74.6	0
T0498-D1	1pgxA	37.8	2qmtA	36.7	2qmtA	0.64	36.7	0
T0499-D1	1pgxA	83.0	1pgxA	83.0	1pgxA	0.64	83.0	0
T0501-D1	1i74A	56.9	1k20A	58.9	1k20A	0.64	58.9	0
T0501-D2	1i74A	31.9	1k20A	55.4	1k20A	0.64	55.4	0
T0502-D1	1ynxA	68.0	1o7iA	73.1	1o7iA	0.64	73.1	0
T0503-D1	2h6bA	54.9	1hw5A	66.0	1hw5A	0.64	66.0	0
T0504-D1 [a]	2g3rA	17.3	2ns2A	44.4	2g3rA	0.64	74.2	29.8
T0504-D2 [a]	2g3rA	48.9	2ns2A	25.6	2g3rA	0.64	32.2	6.6
T0504-D3 [b]	2g3rA	56.1	2ns2A	41.0	2eqmA	0.64	80.7	39.7
T0505-D1	1rkqA	89.0	1rkqA	89.5	1rkqA	0.64	89.5	0
T0505-D2	1rkqA	61.3	1rkqA	63.0	1rkqA	0.64	63.0	0
T0506-D1	2arzA	84.5	2arzA	80.8	2arzA	0.64	80.8	0
T0506-D2	2arzA	77.2	2arzA	79.8	2arzA	0.64	79.8	0
T0507-D1 [c]	1od6A	33.7	1cozA	59.9	1cozA	0.4	62.1	2.2
T0508-D1	1ej0A	84.2	1ej0A	84.5	1ej0A	0.64	84.5	0
T0509-D1	2avdA	72.7	2hnkB	76.9	2hnkB	0.64	76.9	0
T0510-D1 [c]	1tdzA	14.7	1tdzA	16.6	1tdzA	0.8	24.5	7.9
T0510-D2 [c]	1tdzA	48.5	1tdzA	51.8	1tdzA	0.8	67.3	15.5
T0511-D1	2uvdA	71.4	2uvdA	70.0	2uvdA	0.64	70.0	0
T0512-D1	1l0qA	55.6	1l0qA	62.0	1l0qA	0.64	62.0	0
T0513-D1	1vk6A	30.2	2fkbC	48.7	2fkbC	0.64	48.7	0
T0514-D1 [c]	1t1gA	12.3	1ogaD	14.8	1ikpA	0.64	17.5	2.7
Sum		9447.5		9954.4			10331.4	

[a] Template search method contributes for this difference between $(PS)^2$-CASP8 and $(PS)^2$-v2.
[b] Multiple-template method contributes for this difference between $(PS)^2$-CASP8 and $(PS)^2$-v2.
[c] Multiple-model method contributes for this difference between $(PS)^2$-CASP8 and $(PS)^2$-v2.

References

1. Chen CC, Hwang JK, Yang JM: **$(PS)^2$: protein structure prediction server**. *Nucleic Acids Res* 2006, **34**:W152-W157.

2. Chen CC, Yang JM, Hwang JK: **$(PS)^2$: protein structure prediction server**. *Eighth Community Wide Experiment on the Critical Assessment of Techniques for Protein Structure Prediction* 2008:87-88.
