# Supplementary figures and images for: (PS)2-v2: template-based protein structure prediction server
Source: BMC Bioinformatics. 2009 Oct 31;10:366. doi: 10.1186/1471-2105-10-366 (PMC2775752; doi:10.1186/1471-2105-10-366)

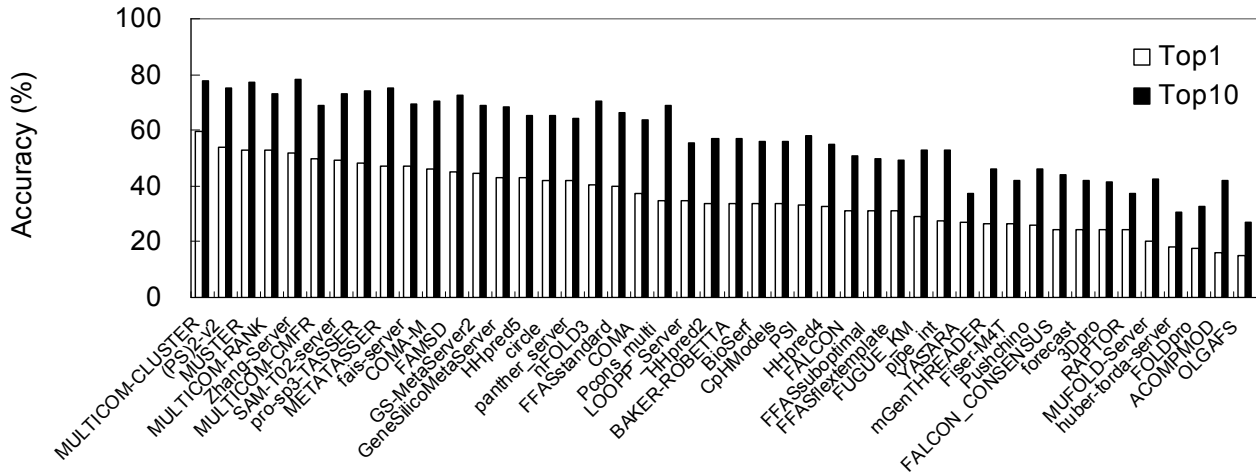

Supplement: Additional file 5 — Figure S2. Comparison of the (PS)2-v2 server with top-ranking 45 servers participating in the CASP8 competition for the template selection on 154 TBM targets. The best templates are directly summarized from the CASP8 website . [file 1471-2105-10-366-S5.pdf]

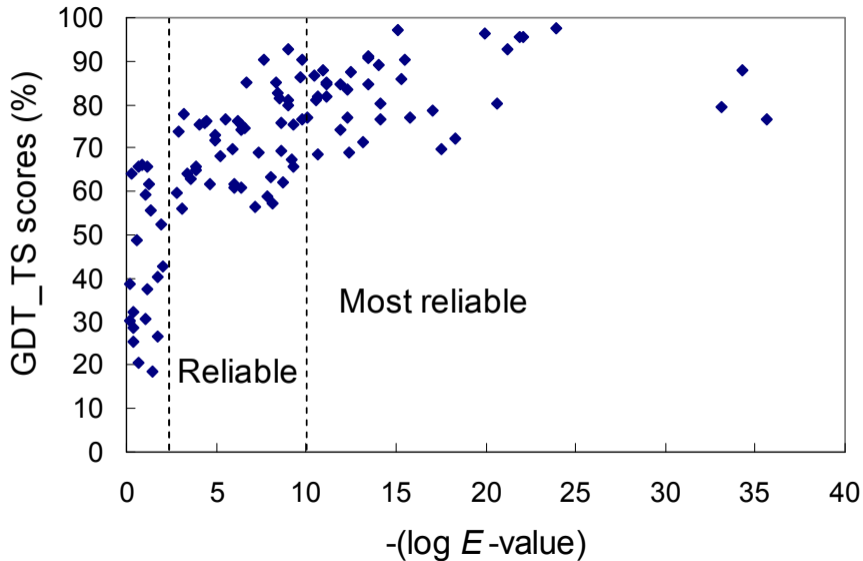

Supplement: Additional file 6 — Figure S3. The relation between E-values and GDT_TS scores of (PS)2-v2 for the targets in CASP8. (PS)2-v2 often yields reliable predicted structures if the E-value is less than 10-2. [file 1471-2105-10-366-S6.pdf]
