# Supplementary material for: (PS)2-v2: template-based protein structure prediction server
Source: BMC Bioinformatics. 2009 Oct 31;10:366. doi: 10.1186/1471-2105-10-366 (PMC2775752; doi:10.1186/1471-2105-10-366)
Supplement: Additional file 7 — Table S3. Comparison of the (PS)2-v2 server and top five servers in CASP8. [file 1471-2105-10-366-S7.pdf]

Table S3. **Comparison of the (PS)^2-v2 server and top five servers in CASP8**

LGA_S [a]/ Methods	Easy > 90% (n[b] =52)		Medium 70% ~ 90% (n=62)		Hard < 70% (n=40)	
	Average [c]	p-value [d]	Average	p-value	Average	p-value
Average of the top 5 servers	84.51	-	68.82	-	47.14	-
(PS)^2-v2	84.11	0.6183	67.57	0.2113	44.21	0.0049

[a] LGA_S score between target and the best template in CASP8.

[b] n is the number of targets.

[c] Average of the GDT_TS score.

[d] The p-value of the paired Student's t-test between (PS)^2-v2 and top-ranking 5 servers.
