# Supplementary material for: (PS)2-v2: template-based protein structure prediction server
Source: BMC Bioinformatics. 2009 Oct 31;10:366. doi: 10.1186/1471-2105-10-366 (PMC2775752; doi:10.1186/1471-2105-10-366)
Supplement: Additional file 8 — Table S4. (PS)2-v2 results for using single-model and multiple-model strategies on 154 targets in CASP8 based on GDT_TS scores. [file 1471-2105-10-366-S8.pdf]

Table S4. **$(PS)^2$-v2 results for using single-model and multiple-model strategies on 154 targets in CASP8 based on GDT_TS scores**

	Number of targets	Number of targets with the same GDT_TS [a]	Number of targets improving GDT_TS by multiple models [b]	Number of targets decreasing GDT_TS by multiple models [c]	Sum of improving GDT_TS by multiple models	p-value
SI [d] ≥ 30%	40	39	0	1	-0.4	0.3235
30%>SI≥20%	47	36	9	2	16.3	0.0231
SI <20%	67	52	14	1	129.4	0.0045
Total	154	127	23	4	145.3	0.0018

[a] The GDT_TS of $(PS)^2$-v2 using multiple-model method equals the one of $(PS)^2$-v2 using single-model method.
[b] The number of targets whose GDT_TS scores are improved by using the multiple-model method
[c] The number of targets whose GDT_TS scores are decreased by using the multiple-model method.
[d] Sequence identity.
