# Supplementary material for: (PS)2-v2: template-based protein structure prediction server
Source: BMC Bioinformatics. 2009 Oct 31;10:366. doi: 10.1186/1471-2105-10-366 (PMC2775752; doi:10.1186/1471-2105-10-366)
Supplement: Additional file 1 — Program. The (PS)2-v2 program. [file 1471-2105-10-366-S1.gz › ps2v2/example/align.html]

Alignment results

```
>2i46A

 Template = 2i46A, Q_len = 181, S_len = 152, Score = 220.9
 Aligned = 87.85%, Identnties = 17.61%, SS_identities = 66.67%

Query:  17 FLQPWIKALIEDNSEHDQYHPSGHVIPSLTKQDLALPHMSPTILTNPCHFAKITKFYNVCDYKVYASIRDSSHQILVEFS 96
           EeChhHHHHccCcCCCCCeeEEEEEEEEEcCCCCCccccccccCCCCCCcCCCCcccccCCCeEEEEEECCCCEEEEEEC
            L+PWI+ LI   SE      +G ++  L   + A        +  P H A  T      D      + D +H +    +
           EcCccHHHHhhC-CCCCCccEEEEEEEEEeCCCCC--------CCCCCC-CCCC-----CCCcEEEEEECCCCEEEEEEC
Sbjct:   5 VLRPWIRELILG-SETPSSPRAGQLLEVLQDAEAA--------VAGPSH-APDT-----SDVGATLLVSDGTHSVRCLVT 69

Query:  97 QECVSNFERTHNCRITSETTNCLMIIGDADLVYVTNSRAMSHFKICLSNISSKEIVPVLNVNQATIFDIDQVGSLSTFP 175
           HHHHHhhhhhCCCeeeCCCCCEEEEEEEEEeEEEECcccccccceeeECccCcCeEEEEEEEEEEEEcccCccCCCCCC
           +E +   +          T   L+++ D   V+V                        L V++ ++   +Q       P
           HHHHHcccccCCCcccCCCCCEEEEEEEEE-EEEEC----------cEC--CeCcEEEEEEEEEEEEeeeC--CCCCCC
Sbjct:  70 REALDTSDWEEKEFGFRGTEGRLLLLQDCG-VHVQV----------AEG--GAPAEFYLQVDRFSLLPTEQ--PRLRVP 133
```
